# Timely Endocytosis of Cytokinetic Enzymes Prevents Premature Spindle Breakage during Mitotic Exit

**DOI:** 10.1371/journal.pgen.1006195

**Published:** 2016-07-22

**Authors:** Cheen Fei Chin, Kaiquan Tan, Masayuki Onishi, YuanYuan Chew, Beryl Augustine, Wei Ren Lee, Foong May Yeong

**Affiliations:** 1 Department of Biochemistry, Yong Loo Lin School of Medicine, National University of Singapore, Singapore; 2 Department of Genetics, Stanford University School of Medicine, Stanford, California, United States of America; Stowers Institute for Medical Research, UNITED STATES

## Abstract

Cytokinesis requires the spatio-temporal coordination of membrane deposition and primary septum (PS) formation at the division site to drive acto-myosin ring (AMR) constriction. It has been demonstrated that AMR constriction invariably occurs only after the mitotic spindle disassembly. It has also been established that Chitin Synthase II (Chs2p) neck localization precedes mitotic spindle disassembly during mitotic exit. As AMR constriction depends upon PS formation, the question arises as to how chitin deposition is regulated so as to prevent premature AMR constriction and mitotic spindle breakage. In this study, we propose that cells regulate the coordination between spindle disassembly and AMR constriction via timely endocytosis of cytokinetic enzymes, Chs2p, Chs3p, and Fks1p. Inhibition of endocytosis leads to over accumulation of cytokinetic enzymes during mitotic exit, which accelerates the constriction of the AMR, and causes spindle breakage that eventually could contribute to monopolar spindle formation in the subsequent round of cell division. Intriguingly, the mitotic spindle breakage observed in endocytosis mutants can be rescued either by deleting or inhibiting the activities of, *CHS2*, *CHS3* and *FKS1*, which are involved in septum formation. The findings from our study highlight the importance of timely endocytosis of cytokinetic enzymes at the division site in safeguarding mitotic spindle integrity during mitotic exit.

## Author Summary

The cytokinesis machinery that is required for physical separation of mother-daughter cells during mitosis is highly conserved from yeast to humans. In budding yeast, cytokinesis is achieved via timely delivery of cytokinetic enzymes to the division site that eventually triggers the constriction of AMR. It has been previously demonstrated that cytokinesis invariably occurs after the disassembly of the mitotic spindle. Intriguingly, Chs2p that is responsible for laying down the primary septum has been shown to localize to the division site before mitotic spindle disassembly. In this study, we show that mitotic spindle integrity upon sister chromatid separation is dependent on the continuous endocytosis of cytokinetic enzymes. Failure in the internalization of cytokinetic proteins during mitotic exit causes premature AMR constriction that eventually contributes to the shearing of mitotic spindle. Consequently, cells fail to re-establish a bipolar spindle in the subsequent round of cell division cycle. Our findings provide insights into how the levels of secreted proteins at the division site impacts cytokinesis. We believe this regulation mechanism might be conserved in higher eukaryotic cells as a secreted protein, hemicentin, has been shown recently to be involved in regulating cytokinesis in both *Caenorhabditis elegans* and mouse embryos.

## Introduction

During mitosis in budding yeast, many cellular processes such as sister chromatid separation and spindle elongation are controlled by the mitotic cyclin-dependent kinase (CDK1) whose activity serves to activate or inactivate its substrates through phosphorylation (reviewed in [1]). As the cell progresses through mitosis, mitotic CDK1 activity is eventually abolished due to the combinatory effect of mitotic cyclins proteolysis and expression of CDK1 inhibitors.

The decline of mitotic CDK1 activity, also known as mitotic exit, is a tightly-regulated process involving components that are highly conserved across species. In eukaryotic cells, destruction of mitotic cyclins depends upon the conserved E3 ubiquitin ligase known as the anaphase promoting complex / cyclosome (APC/C) for ubiquitin-mediated proteolysis by the 26S proteasome [2]. APC/C is activated by two highly conserved proteins, Cdc20p and Cdh1p. The binding of Cdh1p to APC/C is under the control of a Hippo-like signal transduction cascade known as the Mitotic Exit Network (MEN) comprising of Tem1p (a GTPase), Lte1p (a GTP/GDP exchange factor), Cdc15p (Hippo-like kinase), Cdc5p (Polo-like kinase), Dbf2p/Dbf20p (Ser/Thr kinase), Mob1p (a kinase), and its ultimate effector Cdc14p (Ser/Thr phosphatase) [3].

The lowering of mitotic CDK1 activity initiates late mitotic events such as septum formation and cytokinesis. Cytokinesis is the process during which a cell physically cleaves to form two genetically identical progeny cells subsequent to nuclear division. In budding yeast, cytokinesis is accomplished by spatio-temporal coordination of the centripetal deposition of the primary septum (PS) by Chitin Synthase II (Chs2p) and acto-myosin ring (AMR) constriction [4–7]. During mitotic exit, the rough endoplasmic reticulum (RER) export of Chs2p is permitted only in the presence of low mitotic CDK1 activity, which eventually triggers the constriction of the AMR, leading to cytokinesis [8–10]. After completion of PS formation, Fks1p (catalytic subunit of β-1,3-glucan synthase) together with Chs3p (chitin synthase III) synthesizes the glucan-mannan rich secondary septum next to the ingressing PS [6, 11, 12]. These observations are consistent with the idea that Chs2p in budding yeast or β-glucan synthases in fission yeast promote AMR constriction when present at the neck [6, 13].

Interestingly, it has been shown that during normal cell division, Chs2p and Chs3p neck localization precedes mitotic spindle disassembly at late mitosis [7]; Fks1p also localizes to the mother-daughter neck during mitotic exit prior to AMR constriction [14, 15]. Crucially, the decreased mitotic CDK1 activity in late mitosis also promotes mitotic spindle disassembly. Mitotic exit contributes to the dismantling of the mitotic spindles in part by inactivation of mitotic effectors such as those required for spindle elongation [16–18] and in part by targeting the microtubule cross-linking proteins that are involved in mitotic spindle stabilization, such as Cin8p, Ase1p, and Fin1p, for proteaosomal degradation [18–20].

Given that mitotic exit promotes both the neck localisation of cytokinetic enzymes and disassembly of mitotic spindles, the question arises as to how cells ensure that spindles are not broken by premature AMR constriction in a normal cell division due to the activities of cytokinetic enzymes at the bud neck [6, 13]. This is an important issue as cells in which spindle disassembly is delayed have mitotic spindles that are severed as a result of AMR constriction [21]. Indeed, in the absence of Kip3p, a kinesin-8 motor protein that has microtubule depolymerase activity needed to promote microtubule depolymerization during spindle disassembly [21–23], mitotic spindles failed to disassemble in time and were sheared by AMR constriction [21]. This indicates that normally, mechanisms exist to ensure a tight coordination of spindle disassembly and AMR constriction to prevent untimely breakage of the mitotic spindle during mitotic exit.

One relatively-unexplored aspect of the cytokinetic enzymes is how the levels of these enzymes at the neck are regulated during mitotic exit. The timely delivery of cytokinesis enzymes to the neck late in mitosis has been shown to rely upon the secretory pathway trafficking [7, 8, 24, 25]. For instance, Chs2p synthesized at early mitosis is targeted to the neck during mitotic exit when the mitotic CDK1 activity is low [8, 9, 26]. Chs3p and Fks1p are constitutively targeted to the plasma membrane throughout all phases of the cell division cycle [11]. However, the cytokinesis enzymes also accumulate at the neck towards the end of mitosis [14, 15, 27], presumably due to mitotic exit.

At the end of mitosis, clathrin-mediated endocytosis (CME) has been implicated in the removal of Chs2p from the neck [9, 24]. CME is the major route for protein cargo internalization from the plasma membrane in the budding yeast and occurs constitutively [28]. CME is divided into 3 main phases: the early immobile phase, intermediate/late immobile phase, and WASP/ myosin/ actin/ slow mobile invagination phase [reviewed in [29]]. The early immobile phase depends upon a range of proteins including clathrins Chc1p and Clc1p, Eps15 homology (EH) domain protein Ede1p, while the intermediate/late immobile phase relies upon other proteins such as Sla2p and End3p. In the WASP/ myosin/ actin/ slow mobile invagination phase, nucleation promoting factors such as Abp1p are needed to trigger the invagination of the clathrin-coated pit for the internalization of cargoes. Finally, the amphiphysins (Rvs161p/Rvs167p) and dynamin drive the scission of the clathrin vesicle from the plasma membrane by narrowing the neck of the invagination tip. Interestingly, while the forward trafficking of the cytokinetic enzymes to the neck has been fairly-well characterized, the removal of these enzymes by CME especially during late mitosis, has been less so.

As the interplay among septation, AMR constriction, and spindle disassembly is presently poorly understood, we set out to study the mechanisms underlying timely spindle disassembly and cytokinesis. In relation to this, the regulation of the levels of cytokinetic enzymes at the neck during late mitosis was particularly interesting given the relative dearth of information on this aspect. In our present report, we provide evidence that the coordination between mitotic spindle disassembly and AMR constriction is regulated in part via timely endocytosis of cytokinetic enzymes at the division site during mitotic exit. We show using time-lapse fluorescence imaging that during a normal cell division, the cytokinesis enzymes Chs2p, Chs3p, and Fks1p are localized to the mother-daughter neck throughout mitotic exit when the spindles are still intact. Failure to endocytose cytokinetic enzymes at late mitosis results in a thickened cell wall, aberrant septation, and mitotic spindle breakage. Strikingly, when endocytosis of the cytokinetic enzymes is defective, excessive accumulation of cytokinetic enzymes results in premature AMR constriction prior to spindle disassembly. As a consequence of the mitotic spindle breakage, the spindle fails to reassemble in a proportion of cells in the subsequent round of cell division. These findings highlight the vital role of constitutive endocytosis in safeguarding mitotic spindle integrity during cytokinesis.

## Results

### Mitotic spindles are disassembled subsequent to the arrival of cytokinesis enzymes at the neck

To understand how septation, AMR constriction, and mitotic spindle disassembly are coordinated during late mitosis, we used time-lapse microscopy to first characterize the dynamics of cytokinetic enzymes during mitotic exit. We examined the neck localization of cytokinetic enzyme Chs2p-mCherry, and constriction of the Myo1p-GFP ring, relative to mitotic spindle disassembly (visualized by α-tubulin, GFP-Tub1p). The Chs2p-GFP and Myop1-GFP were functional, as W303 cells were inviable without functional Chs2p (S1 Fig) or Myo1p [30].

**Fig 1:**
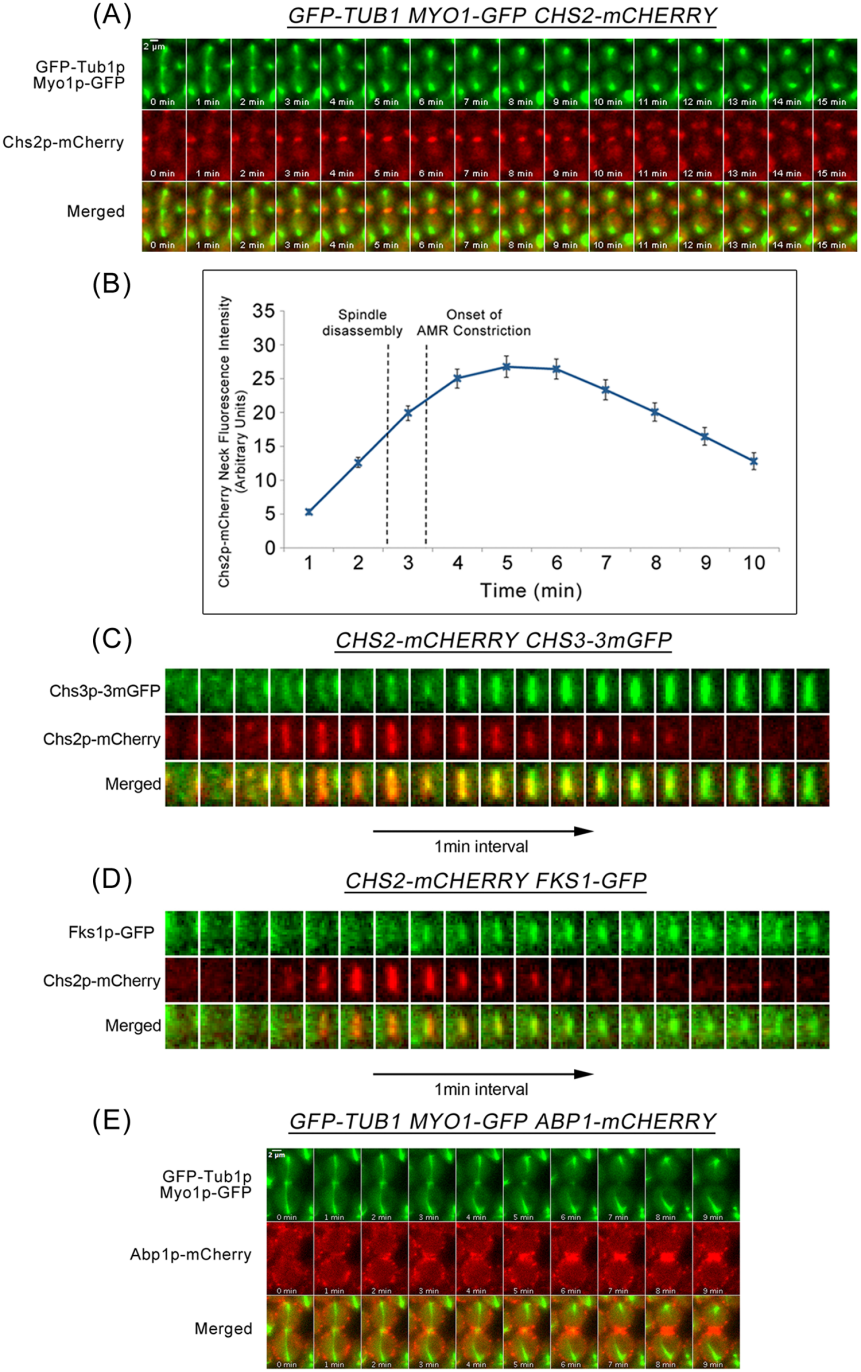
Dynamics of spindle disassembly relative to cytokinetic enzymes’ arrival at the neck and AMR constriction during late mitosis. **(A)** *GFP-TUB1 MYO1-GFP CHS2-mCHERRY* cells were arrested in YPD/Noc at 24°C. After 4 hours, cells were released from metaphase into fresh YPD for 30min. Cells were then mounted in SC/Glu agar pad and examined with time-lapsed microscopy (n = 32). **(B)** Graph showing Chs2p-mCherry neck fluorescence intensity at the neck relative to mean time of spindle disassembly and mean time of AMR constriction. **(C)** *CHS2-CHERRY CHS3-3mGFP* (n = 34), **(D)** *CHS2-CHERRY FKS1-GFP* (n = 27), and **(E)** *GFP-TUB1 MYO1-GFP ABP1-mCHERRY* cells were treated as described above (A) except *FKS1-GFP CHS2-CHERRY* cells were mounted on 0.5x YDP agar pad.

Consistent with results from a previous study [7], in *GFP-TUB1 MYO1-GFP CHS2-mCHERRY* cells released from a Noc arrest, Chs2p-mCherry arrived at the neck 2.06 ± 0.80min before spindle disassembly (2-4min; Fig 1A). The constriction of Myo1p-GFP was initiated at 0.75 ± 0.84min after the disassembly of the mitotic spindle and was completed by 4.44 ± 0.88min (n = 32) (Fig 1A and 1B). This places a time separation between Chs2p neck localization and AMR constriction, with spindle disassembly occurring during the intervening time.

Other than Chs2p that contributes to septation and AMR constriction, other cytokinetic enzymes, Chs3p and Fks1p also play a role in cytokinesis [6, 11, 14]. Though immunofluorescence data from a study by the Pellman group demonstrated that Chs3p-GFP neck signals were observed in cells with intact mitotic spindles labelled by GFP-Tub1p [14], the dynamics of mitotic spindle disassembly relative to the neck localization of other cytokinetic enzymes has not been directly examined. Similarly, Fks1p-GFP has been shown to localize at the bud neck before the onset of AMR constriction [15], though not much is clear about its localisation relative to spindle disassembly.

To determine the dynamics of mitotic spindle disassembly relative to the neck arrival of these other cytokinetic enzymes, we examined the dynamics of Chs3p and Fks1p using Chs2p neck localization as the marker. Both Chs3p-3mGFP (monomeric GFP) and Fks1p-GFP were functional fusion proteins in our strain background (S1 Fig). Chs2p-mCherry neck signals colocalized with Chs3p-3mGFP (0.94 ± 0.85, n = 34) (Fig 1C) and Fks1p-GFP (1.48 ± 1.28, n = 27) (Fig 1D) at the division site. These results suggest that cytokinetic enzymes Chs3p and Fks1p, together with Chs2p, localize to the neck prior to spindle disassembly, while AMR constriction occurs subsequent to spindle disassembly.

The observations together indicate that mechanisms exist to coordinate septation and AMR constriction with spindle disassembly so as to prevent premature breakage of the spindles. Presumably, mitotic exit, a key event that triggers these processes, plays a role in controlling the timing of these events. However, mitotic exit by itself might not be sufficient to prevent untimely septation and constriction of the AMR, given that the cytokinetic enzymes are present at the neck even while the spindles are still intact.

### Accumulation of Chs2p throughout mitotic exit is regulated by CME

A possible mechanism by which cells restrain septation and AMR constriction before spindles disassemble could be through controlling the neck localisation of the cytokinetic enzymes Chs2p, Chs3p and Fks1p [7]. For instance, the levels of these cytokinetic enzymes at the neck could be regulated by altering the rate at which they are removed from the neck via processes such as CME. Indeed, Sla2p, a component of the CME machinery, was reported to play a role in the internalization of Chs2p, albeit at the end of AMR constriction [9, 24]. None the less, given that CME has been previously noted to retrieve cargoes as soon as they arrive at the plasma membrane [31], we sought to characterise the timings of endocytic components appearing at the neck relative to spindle disassembly and the arrival of Chs2p.

We examined the dynamics of key CME proteins that are crucial in facilitating endocytosis during mitotic exit by using Abp1p (actin binding protein that localizes to actin patches) as an endocytosis marker. In *APB1-mCHERRY GFP-TUB1* cells, we noted that Abp1p-mCherry localized to the division site prior to spindle breakage by 1.90 ± 0.99min (n = 70) (Fig 1E). This highlights a likely role of CME in the internalization of Chsp2 at the neck before the end of AMR constriction and raises the possibility of CME being involved in regulating the timing of septation and AMR constriction.

We next investigated the relationship between Chs2p and key CME proteins. In line with the action of Sla2p in Chs2p internalization, Chs2p-mCherry neck localization precedes Abp1p-GFP neck accumulation by 1.9 ± 1.25min, n = 61 (S2E Fig). Similarly, Chs2p-mCherry neck localization also preceded the neck accumulation of all key CME components examined [(Ede1p-GFP, 1.87 ± 0.87min, n = 63), (Sla2p-GFP, 1.52 ± 1.04min, n = 43), (Las17p-GFP, 1.80 ± 0.73min, n = 44), (Sla1p-GFP, 1.33 ± 0.92min, n = 30), and (Rvs167p-GFP, 2.21 ± 0.89min, n = 52)] (S2 Fig). The mass accumulation of endocytic components implies that the rate of endocytosis at the neck increases when cells exit from mitosis.

**Fig 2:**
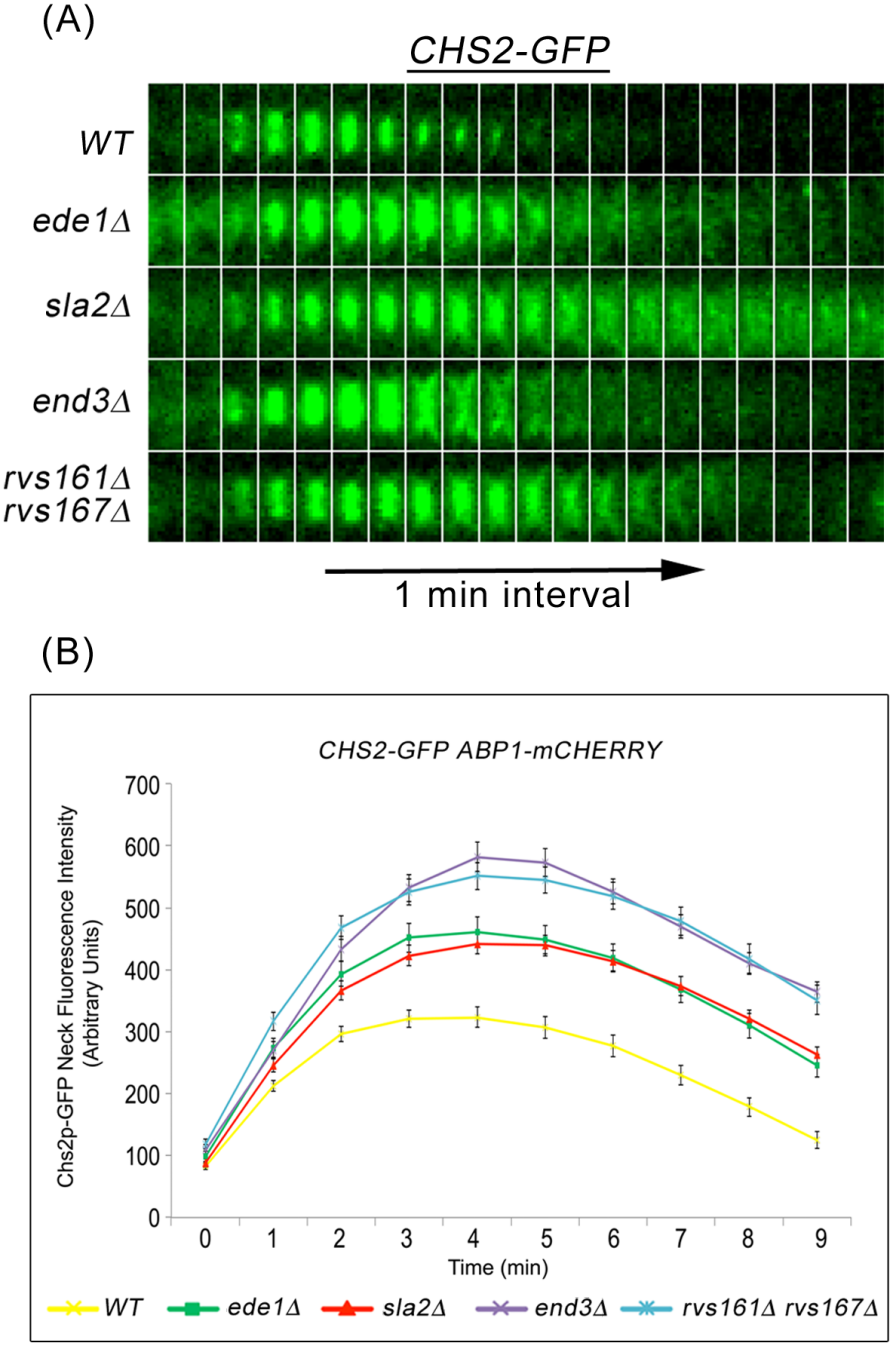
Chs2p-GFP accumulates in endocytosis mutants during mitotic exit. **(A)** *CHS2-GFP ABP1-mCHERRY* cells harbouring deletions of key endocytic components were synchronised in metaphase. After release, cells were mounted on SC/Glu agar pad and examined with time-lapsed microscopy. **(B)** Graph showing Chs2p-GFP mean fluorescence intensity at the neck. Error bars represent SEM.

Accordingly, we determined the effect on Chs2p neck internalization during mitotic exit in mutant cells where endocytosis were defective. To this end, we performed time-lapse fluorescence microscopy of *CHS2-GFP ABP1-mCHERRY* in key endocytosis deletion mutants such as *ede1*Δ, *sla2*Δ, *end3*Δ, and *rvs161*Δ *rvs167*Δ mutants that are defective in different stages of CME [29, 32]. In wild-type cells, Chs2p-GFP localized to and was efficiently internalized from the neck as evident from the disappearance of Chs2p-GFP neck signals (Fig 2A). However, in several key endocytosis deletion mutants, Chs2p-GFP internalization was compromised, as evident from the persistent Chs2p-GFP neck signals (3 min onwards, Fig 2A and 2B). The Chs2p-GFP was initially retained at the division site but slowly diffused to the plasma membrane surrounding the cell (Fig 2A).

To quantify the Chs2p retention at the division site, we measured the fluorescence intensity of Chs2p-GFP. In wild-type cells, the mean intensity of Chs2p-GFP neck signals gradually increased with time but started to decrease 4 min after its arrival at the division site. However, the mean neck fluorescence intensity of Chs2p-GFP in all endocytosis deletion mutants was significantly elevated at all time-points examined, and the neck signals were retained for a longer time as compared to wild-type cells (Fig 2B). Collectively, these data suggest that Chs2p concentration at the neck is likely to be regulated in part by CME during mitotic exit and not merely at the end of AMR constriction.

To determine whether endocytosis of the cytokinesis enzymes depends upon mitotic exit, we tested if Chs2p were internalized when mitotic exit were blocked, such as in metaphase- and telophase-arrested cells. As Chs2p export from the RER to the plasma membrane or neck is sensitive to the mitotic kinase activity [8, 9, 26], we made use of the galactose-inducible Chs2p (6S-6A) mutant where the serine residues phosphorylated by the mitotic CDK were mutated to alanines so that the Chs2p (6S-6A) could exit the RER constitutively even in the absence of mitotic exit [9, 26]. Spc42p-eqFP that is a known central plaque component of the spindle pole bodies (SPBs) [33] was used as a maker for metaphase and telophase arrest.

**Fig 3:**
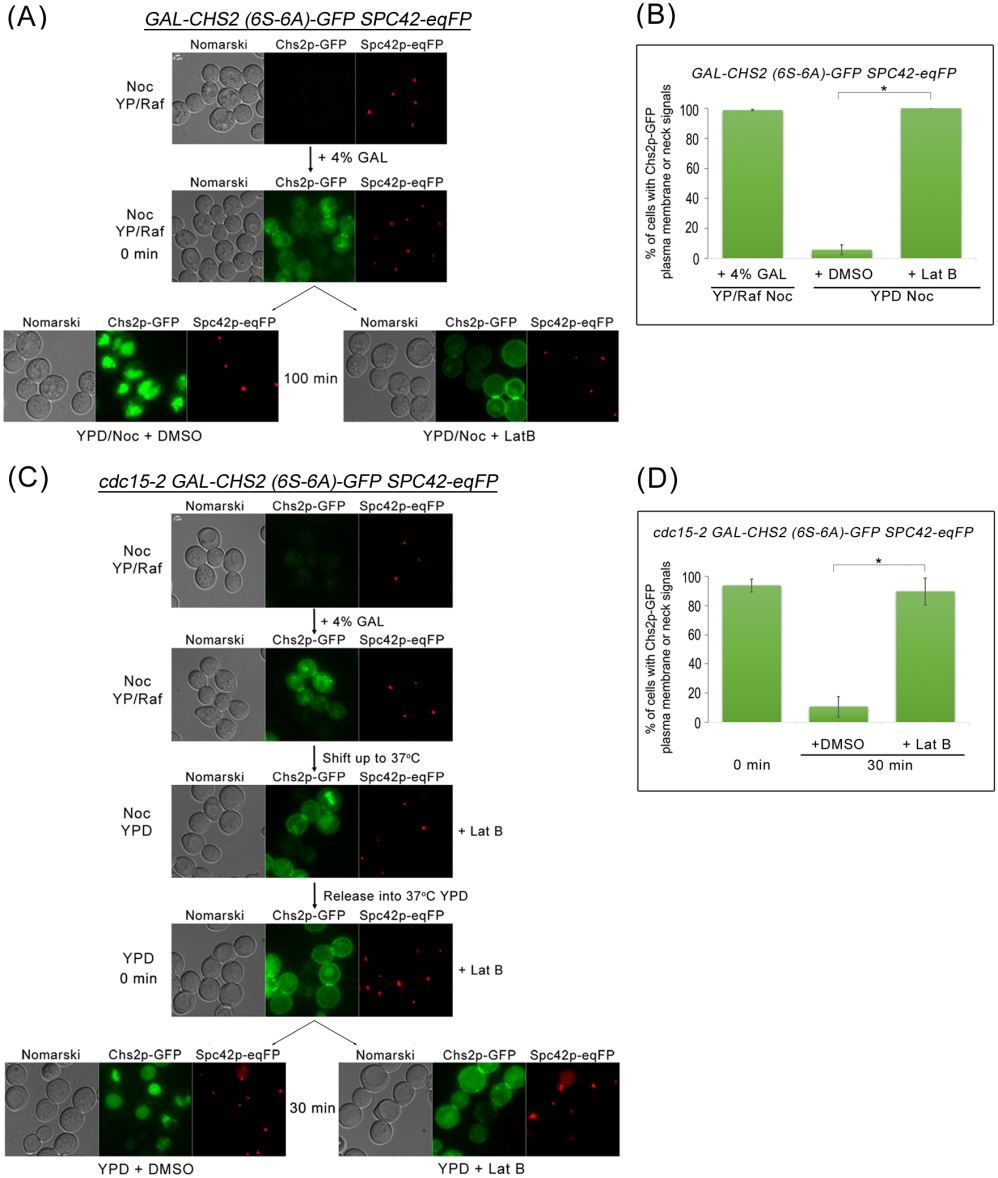
Chs2p-GFP can be endocytosed when mitotic exit is blocked. **(A)** *GAL-CHS2 (6S-6A)-GFP SPC42-eqFP* cells were arrested in YPR/Noc at 24°C. After 5 hours, 4% GAL was added for 60 min. Cells were then split into YPD/Noc contain DMSO or 32μM Lat B, and images were taken after 100 min. **(B)** Graph showing percentage of cells with Chs2p-GFP plasma membrane or neck signals. Error bars represent SD. **(C)** *cdc15-2 GAL-CHS2 (6S-6A)-GFP SPC42-eqFP* cells were arrested in YPR/Noc at 24°C. After 5 hours, 4% GAL was added for 60 min. Cells were then spun into pre-warmed 37°C YPD/Noc with 32μM LatB for 30 min. Next, cells were released into YPD with 32μM Lat B for 60 min, and then culture was split into YPD with DMSO or 32μM Lat B respectively. Images were taken after 30 min. **(D)** Graph showing percentage of cells with Chs2p-GFP plasma membrane or neck signals. Error bars represent SD.

As can be seen, in *GAL-CHS2 (6S-6A)-GFP SPC42-eqFP* cells arrested in metaphase in the presence of Nocodazole (Noc), 98.8 ± 0.5% (n>100) of cells could be observed with neck or plasma membrane Chs2p (6S-6A)-GFP signals due to depolarized transport from the RER to the plasma membrane (including to the neck) in metaphase (Fig 3A and 3B). These Chs2p (6S-6A)-GFP signals converted to vacuolar signals [9] over time (Fig 3A) due to CME-dependent internalisation, indicating that endocytosis occurred even during a metaphase-arrest. Treatment of the cells with a low concentration Latrunculin B (Lat B) that disrupted actin filaments and affected only endocytosis [9] resulted in the accumulation of the Chs2p (6S-6A)-GFP neck or plasma membrane signals (Fig 3A and 3B).

To examine endocytosis of Chs2p (6S-6A)-GFP during a late mitotic exit block, we used the *cdc15-*2 allele that prevents the complete destruction of the mitotic CDK activity when cultured at the restrictive temperature of 37°C [34]. *cdc15-*2 cells typically arrest at 37°C in telophase with high mitotic CDK activity [35, 36]. In *GAL-CHS2 (6S-6A)-GFP SPC42-eqFP cdc15-*2 cells arrested at 37°C, we observed that Chs2p (6S-6A)-GFP was internalized (Fig 3C and 3D). Again, the endocytosis of Chs2p (6S-6A)-GFP was abolished in the presence of sub-optimal concentration of Lat B (89.6% ± 0.5%, n>100; Fig 3D). Our data showing that Chs2p is endocytosed during a block in mitotic exit imply that endocytosis of cargoes at the plasma membrane can occur independently of mitotic exit and is consistent with previous reports showing the recruitment of endocytosis components by the presence of cargoes [31]. More importantly, the data support the notion that cytokinetic enzymes can be endocytosed upon arrival at the neck as the endocytosis machinery appears to function constitutively.

### Chs3p and Fks1p are also internalised via CME during mitotic exit

In wild-type cells shortly after the primary septum is laid down, Chs3p and Fks1p deposit the secondary septum on either side of the primary [4]. Also, in *chs2*Δ mutant cells where the PS is absent, a remedial septum is laid by Chs3p [6]. We therefore asked whether both Chs3p and Fks1p are also regulated in a manner similar to Chs2p at the end of mitosis.

**Fig 4:**
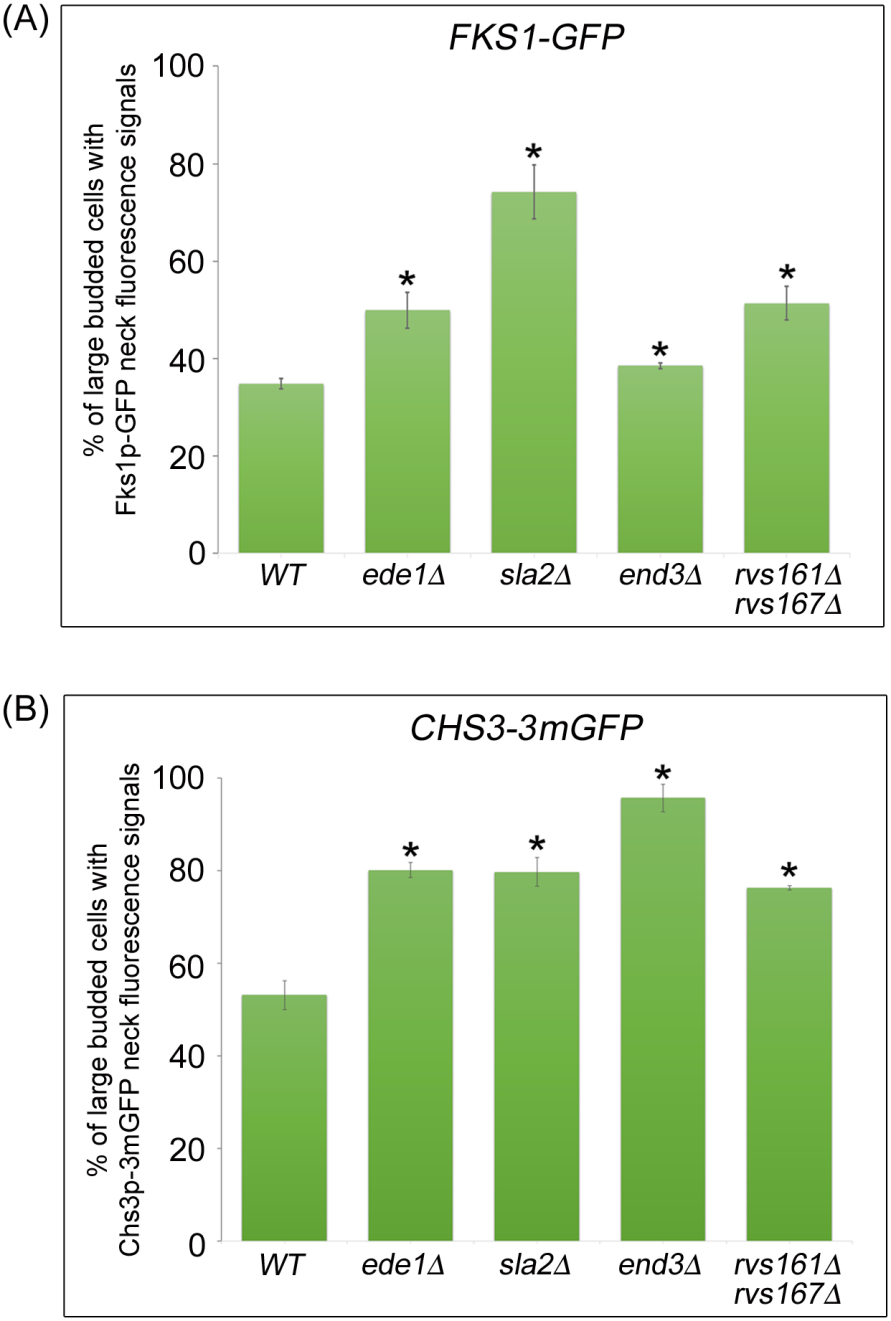
Cytokinetic enzymes Chs3p and Fks1p accumulate in endocytosis-defective mutants. **(A)** *FKS1-GFP ABP1-mCHERRY* and **(B)** *CHS3-3mGFP ABP1-mCHERRY* cells harbouring deletions of key endocytic components were cultured in YPD at 32°C for 2 hours. Cells were then examined under fluorescence microscope. Error bars represent SD.

Unlike Chs2p that is specifically expressed during mitosis, Fks1p is constitutively expressed throughout the cell division cycle. Upon synthesis in the RER, Fks1p is delivered to the plasma membrane via the secretory pathway in a polarized fashion [25]. Similar to Chs2p, Fks1p is subsequently transported to the vacuole for degradation [37]. To study the role of endocytosis in regulating Fks1p localisation at the division site during mitotic exit, we examined the neck localization of Fks1p-GFP in large-budded cells isolated from cycling culture at 32°C. In wild-type cells, Fks1p-GFP neck localization was observed in 34.8 ± 1.0% of large-budded cells. Strikingly, we found that the incidence of Fks1p-GFP neck signals was significantly higher in all endocytosis mutants analyzed as compared to wild-type cells [*ede1*Δ = 49.9 ± 3.7%, *sla2*Δ = 74.2 ± 5.5%, *end3*Δ = 38.5 ± 0.6%, *rvs161*Δ *rvs167*Δ = 51.3 ± 3.4%, (n>600)] (Fig 4A). These results suggest that the levels of Fks1p at the division site might be regulated through CME as defects in endocytosis contribute to abnormal Fks1p neck accumulation.

In contrast to Chs2p and Fks1p that are subjected to vacuolar degradation upon arrival at the neck, Chs3p is not targeted to vacuoles for degradation. Rather, it is recycled between the plasma membrane and the chitosome [24, 37, 38]. Temperature-sensitive mutations that block endocytosis, *end3-1* and *end4-1*, resulted in a reduction of Chs3p levels in chitosomes, suggesting that Chs3p levels at the plasma membrane are regulated via endocytosis [24]. We speculated that defective endocytosis might also lead to premature accumulation of Chs3p at the mother-daughter neck.

We therefore compared bud-neck localization of Chs3-3mGFP during mitotic exit between large-budded cells in asynchronously growing wild-type and endocytosis deletion mutant cells in the manner as described for Fks1p-GFP (Fig 4A). In wild-type cells, about 53.1% of large-budded cells showed Chs3p-3mGFP neck signals. As anticipated, we found that the percentage of Chs3p-3mGFP neck signals in endocytosis deletion mutant cells was significantly higher in comparison with wild-type cells [*ede1*Δ = 79.7 ± 3.1%, *sla2*Δ = 95.7 ± 3.0%, *end3*Δ = 80.1 ± 1.7%, *rvs161*Δ *rvs167*Δ = 76.3 ± 0.5%, (n>500)] (Fig 4B). This result implies that endocytosis also plays an important role in the retrieval of Chs3p from the neck during mitosis. Collectively, our results suggest that the levels of the cytokinetic enzymes, Chs2p, Fks1p, and Chs3p are in part regulated by CME at the end of mitosis.

Based on the importance of CME at the end of mitosis, we next determined the effect of compromised endocytosis on the cell wall, given that ultrastructure of the septum in key endocytosis deletion mutants has not been documented except for the temperature sensitive mutant *sla2-41* [39]. In agreement with our fluorescence microscopy data showing accumulation of Chs2p, Chs3p, and Fks1p, the cell wall of the *end3*Δ and *sla2*Δ mutants formed extra layers of chitin-rich primary septum and abnormally thick secondary septa when examined using transmission electron microscopy (S3 Fig). These results suggest that the thickened septum observed in endocytosis mutants is perhaps due to a failure in the retrieval of chitin synthases and glucan synthase at the division site. Taken together, the data imply that endocytosis at the division site is important for the removal of excessive cytokinetic enzymes to prevent aberrant cell wall formation and abnormal septation during mitosis.

### Mitotic spindle breakage occurs during mitotic exit in key endocytosis deletion mutants

Given that defective endocytosis of cytokinesis enzymes leads to irregular chitin deposition, we wondered if there were any defects in AMR constriction or spindle dynamics as these enzymes normally arrive at the neck prior to disassembly of the mitotic spindles. We first examined if there were defects in the progression of mitotic exit in the endocytosis mutants. From western blot analyses, we noted that mitotic exit in the endocytosis defective cells was comparable to that in wild-type cells (S4 Fig). We then examined AMR constriction and spindle dynamics in endocytosis mutants harboring *GFP-TUB1 MYO1-GFP*.

**Fig 5:**
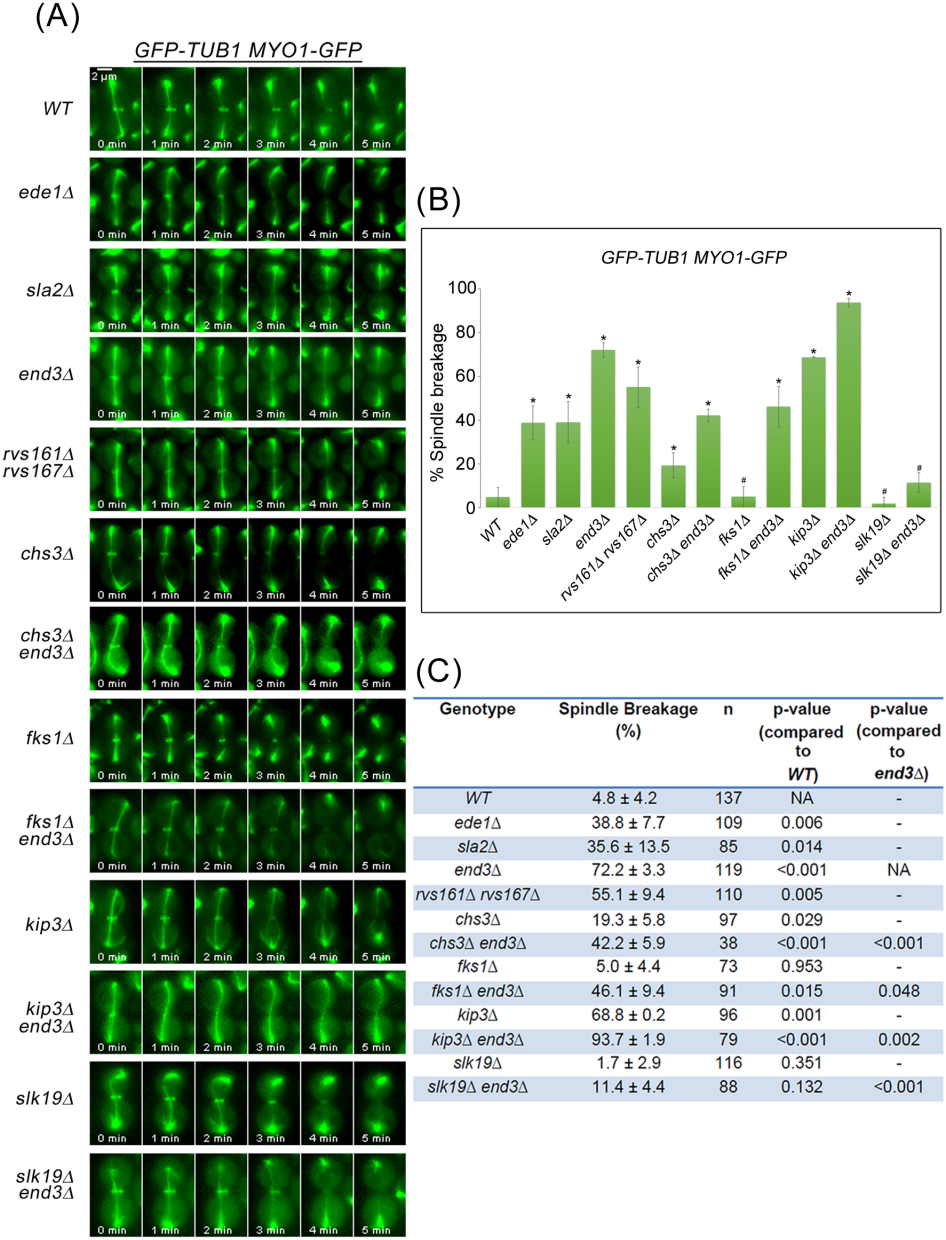
Mitotic spindles appear to break during mitotic exit in key endocytosis deletion mutants. **(A)** *GFP-TUB1 MYO1-GFP* cells harbouring deletions of key endocytic components were synchronised in metaphase and then shifted up to 32°C for 30min. After release, cells were mounted on SC/Glu agar pad and examined with time-lapsed microscopy. **(B)** Graph showing percentage of spindle breakage in endocytosis mutants. Asterisk represents statistical significance as compared to wild-type cells and hash represents no statistical significance as compared to wild-type cells (Student-t test). Error bars represent SD. **(C)** Table showing the percentage of spindle breakage and p-values for endocytosis mutants.

Consistent with results from a previous study [21] and our data above (Fig 1A), the mitotic spindle disassembled prior to constriction of Myo1p-GFP in wild-type cells (95.2% ± 4.2%, n = 137) (Fig 5A, 5B and 5C). In contrast, all of the endocytosis deletion mutants examined displayed varying degrees of what appeared to be mitotic spindle breakage as evident from the execution of AMR constriction prior to mitotic spindle disassembly. Indeed, in 38.8 ± 7.7% of *ede1*Δ (n = 109), 35.6 ± 13.5% of *sla2*Δ (n = 85), 72.2 ± 3.3% of *end3*Δ (n = 119) and 55.1 ± 9.4% of *rvs161*Δ *rvs167*Δ (n = 110) cells, AMR constriction occurred before mitotic spindle disassembly (Fig 5A, 5B and 5C), resulting in possible spindle breakage.

**Fig 6:**
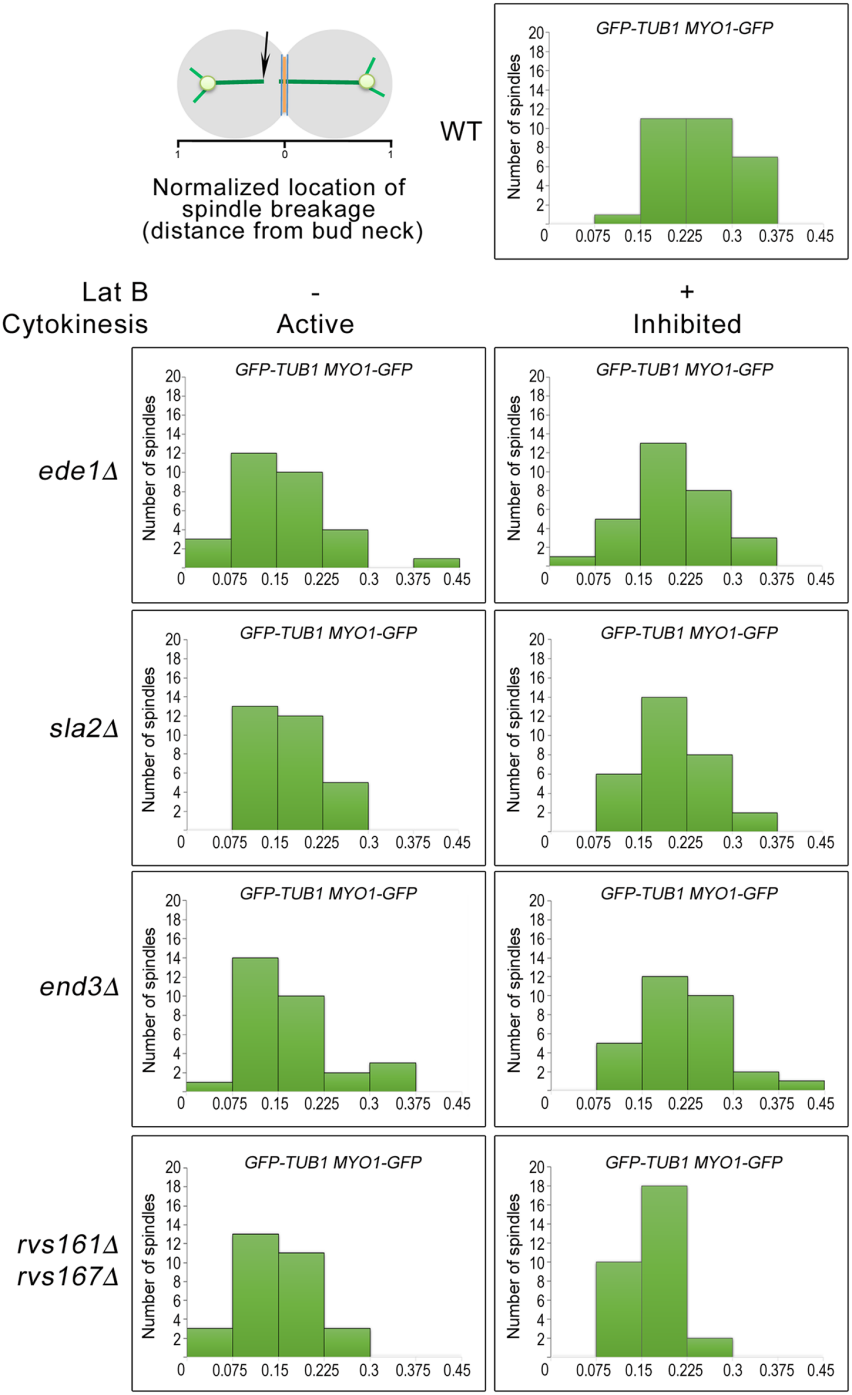
Uneven distribution of initial location of spindle-halves can be observed in endocytosis mutants undergoing mitotic exit. *GFP-TUB1 MYO1-GFP* cells harbouring deletions of key endocytic components were synchronised in metaphase. After 4 hours, 200μM Lat B was added and cells were shifted to 32°C for 30min. Next, cells were released into fresh YPD containing 200μM Lat B for 30 min. Cells were then mounted on SC/Glu agar pad containing 200μM Lat B and subjected to time-lapsed microscopy. The distance of spindle breakdown relative to the bud neck was measured and normalized with cell length as described in Woodruff et al., 2010 (n = 30 for each strain).

To investigate the possibility of spindle breakage in the endocytosis mutants at a greater detail, we observed the initial location of spindle-halves separation during spindle breakdown in wild-type and endocytosis mutant cells as previously described [21]. When the various strains harbouring *GFP-TUB1 MYO1-GFP* were released from Noc into fresh medium containing DMSO, the endocytosis mutants mostly exhibited an uneven distribution of the initial location of spindle-halves separation, with a population located nearer the bud neck. This was unlike the wild-type cells where the initial location of spindle-halves separation was widely distributed (Fig 6). This implies that the spindles in the endocytosis were indeed sheared by AMR constriction instead of undergoing normal spindle disassembly. In support of this idea, when the strains were released from Noc into medium containing Lat B that inhibited AMR constriction, the distributions of the initial location of spindle-halves separation in the endocytosis mutants became more widely distributed (Fig 6).

**Fig 7:**
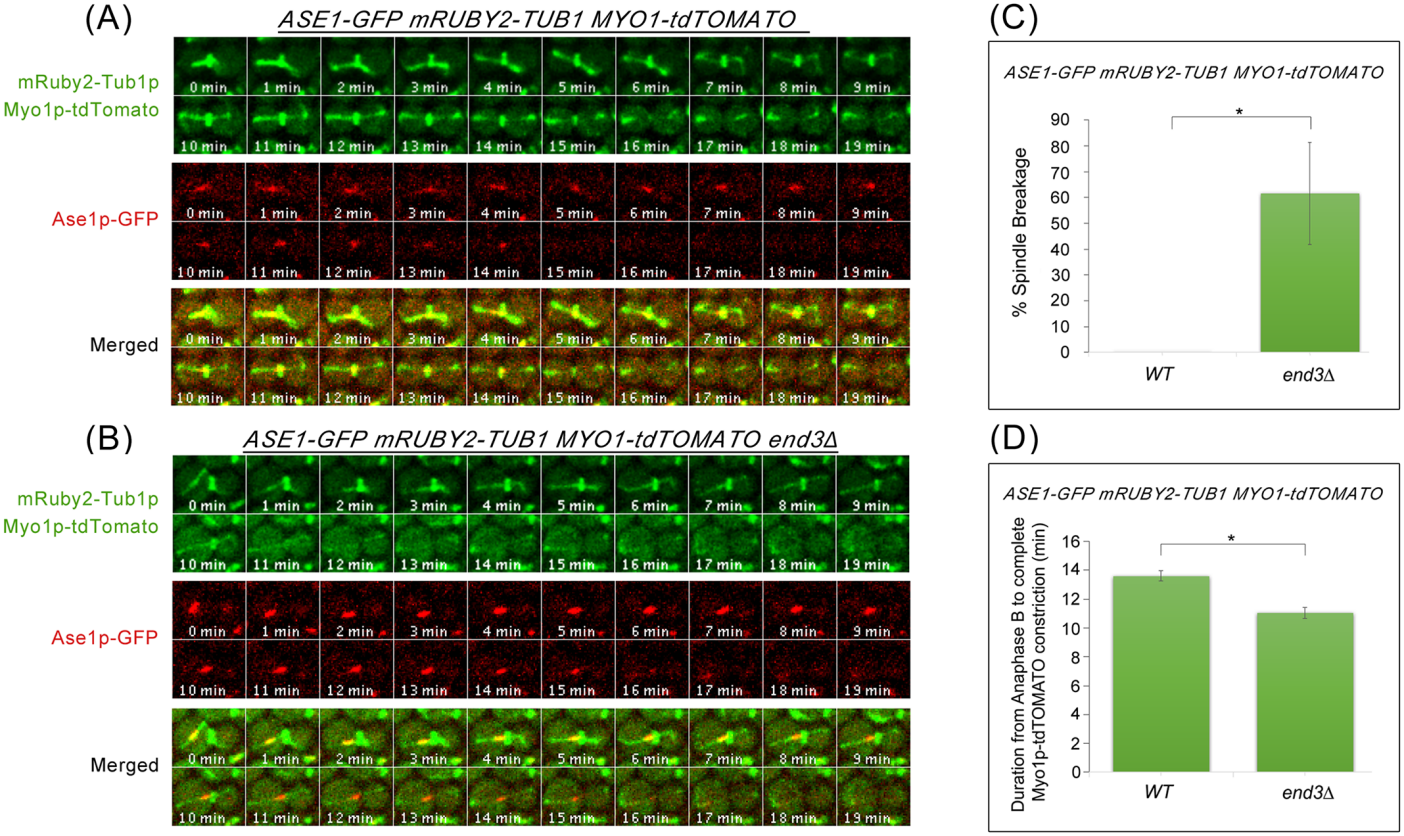
*end3*Δ cells show shorter interval between anaphase B and Myo1p-GFP constriction. **(A)** Wild Type and **(B)** *end3*Δ cells harbouring *mRUBY2-TUB1 MYO1-tdTOMATO ASE1-GFP* respectively were synchronised in metaphase and then shifted up to 32°C for 30min. After release, cells were mounted on SC/Glu agar pad and examined with time-lapsed microscopy. **(C)** Graph showing percentage of spindle breakage in WT and *end3*Δ respectively (WT = 44, *end3*Δ = 43). Error bars represent SD. **(D)** Graph showing duration from anaphase B to Myo1p-tdTOMATO complete constriction (Student t-test, p<0.001; error bars represents SEM).

As a further confirmation of the spindle breakage in the endocytosis mutants, we made use of a midzone marker Ase1p-GFP to analyse the ends of spindle-halves as cells exited from mitosis. *ASE1-GFP mRUBY2-TUB1 MYO1-tdTOMATO* and *ASE1-GFP mRUBY2-TUB1 MYO1-tdTOMATO end3*Δ cells were examined following release from Noc arrest. *end3*Δ was chosen as a representative endocytosis mutant as it is a key CME component. As can be seen, in wild-type cells, Ase1p-GFP decorates the midzone during the onset of anaphase, and dissociates from the spindle prior to constriction of AMR. (Fig 7A). In *end3*Δ cells, we noticed the spindle was sheared by AMR in the region outside the midzone and Ase1p-GFP remained on the broken spindle halves (12min; Fig 7B). 61.6 ± 19.7% of *end3*Δ cells (n = 43) exhibited broken spindle-halves, which was significantly higher than in wild-type cells (n = 44) (Fig 7C). In addition, when we measured the time from anaphase B (spindle length >6μm) to the completion of Myo1p-GFP constriction, we found a shorter interval in *end3*Δ cells as compared to wild-type cells (Fig 7D). Collectively, the data hint at a loss of coordination between spindle disassembly and AMR constriction in the endocytosis mutants, leading to the shearing of the mitotic spindles.

### Premature AMR constriction in endocytosis mutants causes spindle breakage during mitotic exit

**Fig 8:**
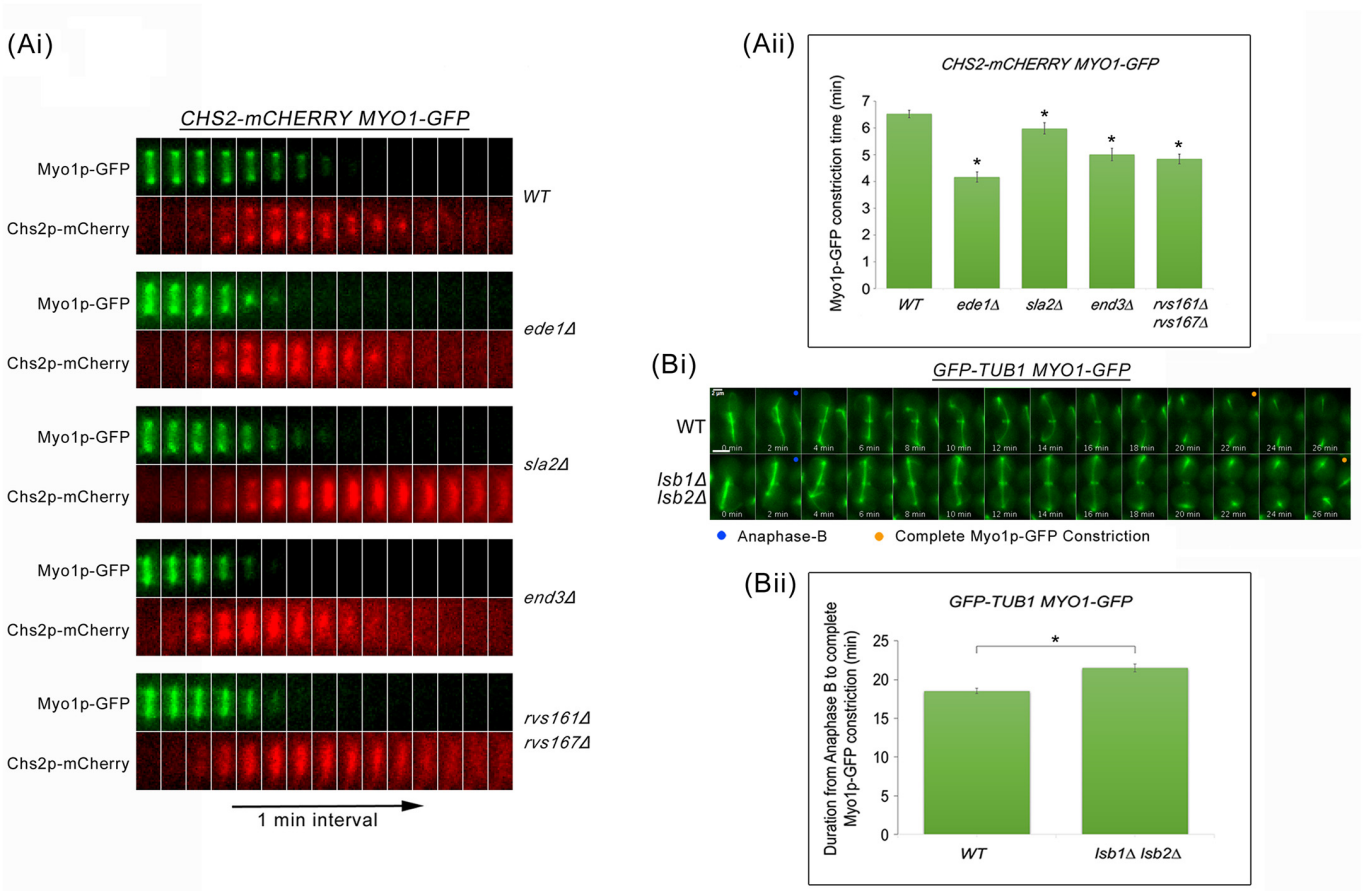
Premature AMR constriction in endocytosis mutants causes spindle breakage during mitotic exit. **(Ai)** *CHS2-mCHERRY MYO1-GFP* cells harbouring deletions of key endocytic components were synchronised in metaphase and then shifted up to 32°C for 30min. After release, cells were mounted on SC/Glu agar pad and examined with time-lapsed microscopy. **(Aii)** Graph showing time taken for Myo1p-GFP complete constriction upon Chs2p-mCherry neck signal arrival. Statistical analysis via Mann Whitney-U test. Error bars represent SEM. **(Bi)** Wild Type and *lsb1*Δ *lsb2*Δ cells harbouring *GFP-TUB1 MYO1-GFP* were arrested in metaphase. After release, cells were mounted on SC/Glu agar pad and subjected to time-lapsed microscopy at 24°C, at 1min intervals. **(Bii)** Graph showing duration from anaphase B to Myo1p-GFP complete constriction (Student t-test, p<0.001). Error bars represent SEM (n = 60).

To test the idea that excessive cytokinetic enzymes in endocytosis mutants might trigger premature AMR constriction leading to the shearing of the mitotic spindles, we next examined the dynamics of AMR constriction by measuring the time taken for complete constriction of Myo1p-GFP relative to Chs2p-mCherry neck localization in wild-type and key endocytosis deletion mutants (Fig 8Ai and 8Aii). In wild-type cells, the constriction of AMR was completed 6.52 ± 0.13min (n = 50) after Chs2p arrival. As anticipated, all key endocytosis deletion mutants showed an accelerated AMR constriction in comparison to wild-type cells. The times taken for Myo1p-GFP constriction upon Chs2p-mCherry arrival in *ede1*Δ (4.16 ± 0.19min, n = 50), *sla2*Δ (5.98 ± 0.21min, n = 50), *end3*Δ (5.0 ± 0.23min, n = 50), and *rvs161*Δ *rvs167*Δ (4.84 ± 0.18min, n = 50) cells were significantly shorter as compared to wild-type cells (p-value <0.0001) (Fig 8Aii). The observations support the notion that failure in CME internalization of cytokinetic enzymes might trigger premature septum deposition and consequently, premature AMR constriction.

Given that endocytosis defects can cause premature AMR constriction, we tested whether increasing the efficiency of endocytosis would lead to the opposite effect. To this end, we released *GFP-TUB1 MYO1-GFP* and *lsb1*Δ *lsb2*Δ *GFP-TUB1 MYO1-GFP* cells from Noc arrest and performed time-lapse imaging to examine the dynamics of AMR constriction relative to anaphase B. *LSB1* and *LSB2* encode for the inhibitors of Las17p [40, 41] and were deleted to increase the internalization of cytokinetic enzymes. In comparison to wild-type cells, *lsb1*Δ *lsb2*Δ cells exhibited a longer duration of AMR constriction (Fig 8Bi and 8Bii), supporting the idea that endocytosis of cytokinetic enzymes can influence AMR constriction. This is in line with the hypothesis that endocytosis plays a role in the neck levels of cytokinesis enzymes, which then influences the dynamics of AMR constriction.

As premature AMR constriction can lead to spindle breakage, we next tested the idea that perturbing the coordinated occurence of AMR constriction relative to spindle disassembly alters the incidences of spindle breakage. On the one hand, we made use of the *kip3*Δ mutant that failed to dismantle spindles in a timely manner [21, 23] in combination with the *end3*Δ. As can be seen, the percentage of spindle breakage in the *end3*Δ mutant cells was increased with deletion of *KIP3* (Fig 5A, 5B and 5C) to 93.7 ± 1.9% (n = 79).

On the other hand, when we destabilized spindles in the *end3*Δ mutant by deleting *SLK19* (microtubule stabilizing protein) [42], the double mutant had greatly reduced the spindle breakage of 11.4 ± 4.4% (n = 88) (Fig 5B and 5C). This data supports the notion that a loss of coordination in the relative timing of AMR constriction and spindle disassembly results in spindle breakage in *end3*Δ cells. The results further imply that the timely turnover of cytokinetic enzymes at the neck in a continuous CME dependent manner plays a role in restraining AMR constriction prior to spindle disassembly.

### Spindle breakage phenotype in *end3*Δ can be rescued by deletion of *chs2*, *chs3* and *fks1*

We therefore asked if the deletion of the genes encoding the cytokinesis enzymes would rescue the spindle breakage phenotype in *end3*Δ cells. As a control, we used *kip3*Δ cells that were shown previously to exhibit spindle breakage phenotype [21]. The phenotype was attributed to Kip3p being a kinesin-8 motor protein that promotes microtubule depolymerization during spindle disassembly [21, 23], and that failure to disassembly spindle in a timely manner results in spindle breakage during AMR constriction in *kip3*Δ cells. In our setup, 68.8 ± 0.2% of *kip3*Δ cells exhibited spindle breakage, in agreement with a previous report [21]. The spindle breakage in the *end3*Δ mutant could be rescued by deleting *CHS3* as the spindle breakage percentage in the *chs3*Δ *end3*Δ double mutant dropped to 42.2 ± 5.9% (n = 38) from 72.2 ± 3.3 (n = 119) in the *end3*Δ mutant (Fig 5B and 5C). Surprisingly, deleting *FKS1* also rescued spindle breakage in the *fks1*Δ *end3*Δ double mutant (46.1 ± 9.4%, n = 91) (Fig 5B and 5C), suggesting that Fks1p plays a far bigger role in secondary septum formation than previously described.

The role of Chs2p in rescuing the *end3*Δ spindle breakage phenotype could not be assessed using *chs2*Δ cells due to compromised viability of the *chs2* null mutant in the W303 background [43]. To overcome this issue, we utilized an improved version of auxin-inducible-degron system for rapid depletion of Chs2p during mitotic exit. We constructed the auxin-degradable *CHS2* strain by fusing a degron containing single copy of mini-AID [minimum region of IAA17 (65–132 amino acids) required for degradation] to the C-terminal end of *CHS2*. In addition, the yeast-codon-optimized *Oryzae sativa* E3 ubiquitin ligase under the *ADH1* promoter (pADH1-*yeOSTIR1*) was integrated at the *LEU2* locus [44, 45].

To determine the role of Chs2p in shearing of mitotic spindle during mitotic exit [44, 45], *END3* or *end3*Δ cells harbouring *GFP-TUB1 MYO1-GFP CHS2-*1xMini-AID were examined in cells release from Noc arrest. Indole-3-acetic acid (IAA) was added to induce Chs2p degradation before cells were released into YPD containing IAA. In our experiments, a sub-optimal concentration of IAA, 0.25mM was used, as complete depletion of Chs2p will cause the breakage of AMR, leading to cytokinesis defects (S5 Fig) [7].

**Fig 9:**
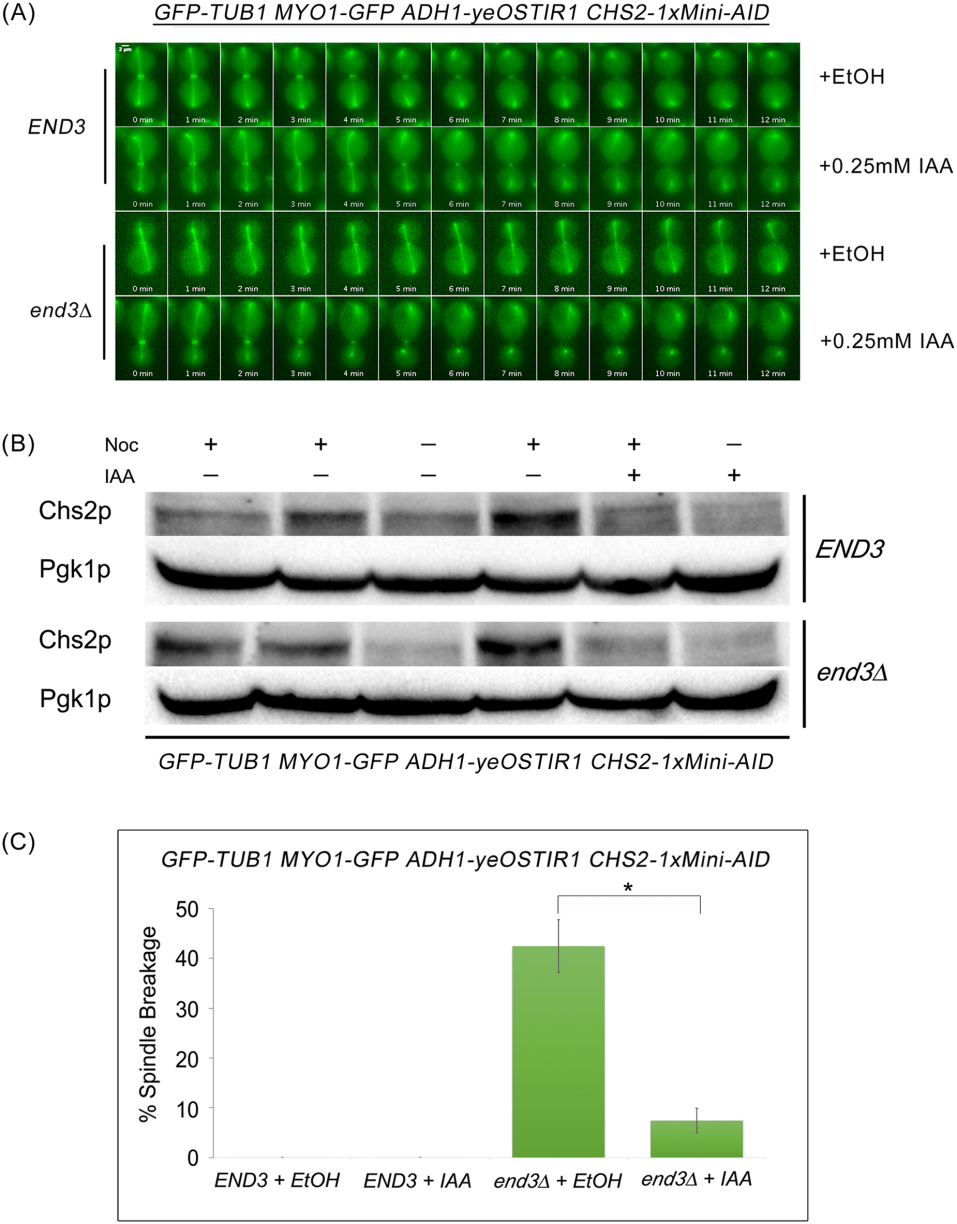
Mitotic spindle breakage phenotype in *end3*Δ cells can be rescued by depleting Chs2p. **(A)** Wild Type and *end3*Δ cells habouring *GFP-TUB1 MYO1-GFP ADH1-yeOSTIR1 CHS2-1xMini-AID* were synchronised in metaphase. After 4 hours, IAA was added to final concentration of 0.25mM and cells were shifted to 32°C for 30min. Next, cells were released from metaphase into fresh YPD containing 0.25mM IAA for 30min. Cells were then mounted on SC/Glu agar pad containing 0.25mM IAA and subjected to time-lapsed microscopy. **(B)** Immunoblot showing degradation of Chs2p following treatment with IAA. Samples were collected at metaphase arrest, after addition of IAA and shifting to 32°C for 30min, and before time-lapse microscopy. **(C)** Graph shows percentage of spindle breakage in WT and *end3*Δ cells that were not treated and treated with IAA respectively. Error bars represent SD (n>100).

In *END3* cells, mitotic spindle breakage was not observed either with (n = 240) or without IAA (n = 217) (Fig 9A and 9C). Conversely, *end3*Δ cells that were not treated with IAA displayed a mitotic spindle breakage phenotype with a percentage breakage of 42.4 ± 5.3% (n = 139) (Fig 9A and 9C). The shearing of mitotic spindle was greatly reduced to 7.4 ± 2.5% (n = 121) when Chs2p concentration was depleted using IAA (p-value< 0.001) (Fig 9B and 9C). Although sub-optimal concentration of IAA was used to deplete Chs2p level, we also observed some cells exhibited breakage of AMR; the cells were excluded from our analysis. The results suggest that levels of Chs2p at the neck is a key determining factor that contributes to mitotic spindle breakage in endocytosis mutants.

### Suppression of cytokinetic enzymes activity rescues spindle breakage in the *end3*Δ mutant

**Fig 10:**
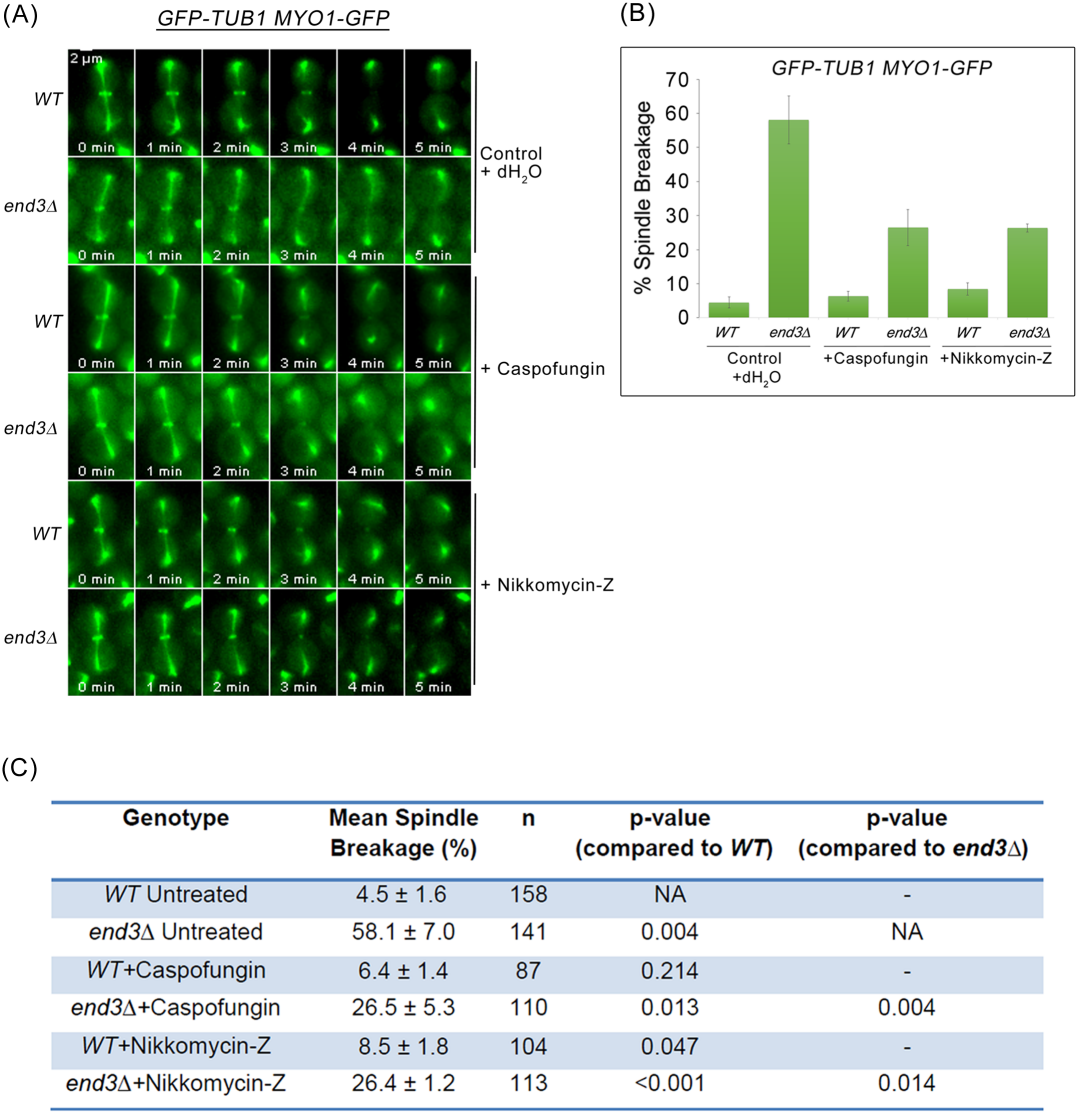
Suppression of cytokinetic enzymes activity via treatment with Caspofungin and Nikkomycin-Z rescues spindle breakage in *end3*Δ mutant. **(A)** *GFP-TUB1 MYO1-GFP* and *GFP-TUB1 MYO1-GFP end3*Δ cells were arrested in YPD/Noc at 24°C for 2 hours. Cells were treated with 50ng/ml caspofungin and 1mM nikkomycin-Z respectively for another 2 hours in YPD/Noc. Upon arrested in metaphase, cells were shifted to 32°C for another 30min. Next, cells were released from metaphase into fresh YPD for 30min. Cells were then mounted in SC/Glu agar pad and examined with time-lapsed microscopy. **(B)** Graph showing the mean percentage of cells with spindle breakage. Error bars represent SD. **(C)** Table showing the mean percentage of cells with spindle breakage and p-values of statistical analysis (Student-t test).

To confirm that the cytokinetic enzymes were indeed responsible for triggering premature AMR constriction, Myo1p-GFP and GFP-Tub1p were observed in endocytosis mutants while the activities of Chs3p or Fks1p were inhibited using nikkomycin-Z (chitin synthase III specific inhibitor) [46] and caspofungin [β(1–3)-D-glucan synthase inhibitor] [47], respectively (Fig 10A). The incidences of spindle breakage of the caspofungin and nikkomycin-Z treated *end3*Δ mutant cells was decreased to 26.5 ± 5.3% (n = 110) and 26.4 ± 1.2% (n = 113) respectively (Fig 10B and 10C). The combinatorial use of nikkomycin and caspofungin was not possible as cells were not viable even when treated acutely. The reduction in spindle breakage incidences in *end3*Δ mutant cells treated separately with nikkomycin-Z or caspofungin supports the notion that accumulation of cytokinesis enzyme activities led to premature AMR constriction dynamics and untimely spindle breakage.

**Fig 11:**
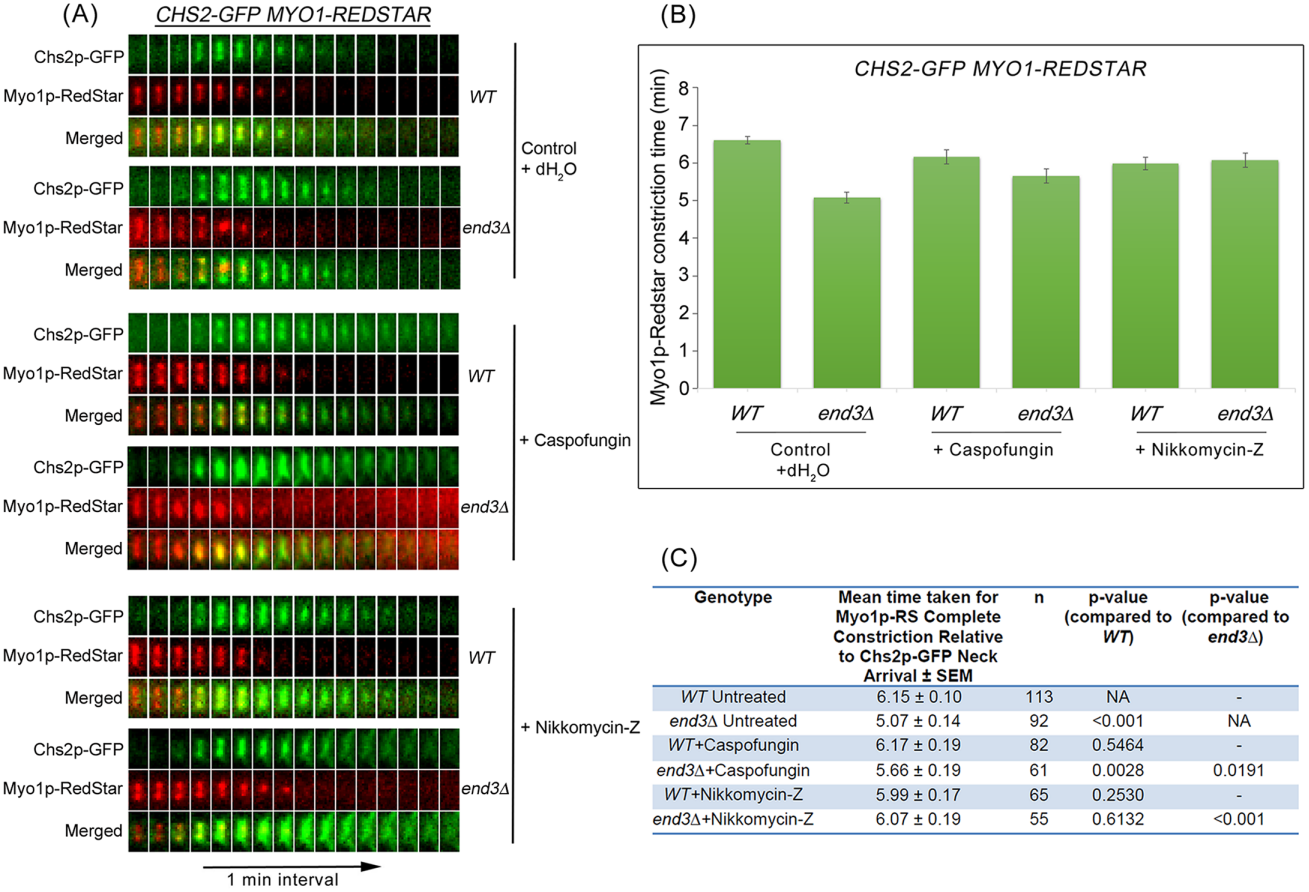
Suppression of cytokinetic enzymes activity via treatment with Caspofungin and Nikkomycin-Z alters AMR constriction dynamics. **(A)** *CHS2-GFP MYO1-REDSTAR* and *CHS2-GFP MYO1-REDSTAR end3*Δ cells were treated as in (Fig 10A). **(B)** Graph showing time taken for complete AMR constriction relative to Chs2p-GFP neck localization. Error bars represent SEM. **(C)** Table showing the mean time taken for complete Myo1p-GFP constriction relative to Chs2p-GFP neck localization and p-values of statistical analysis (Mann Whitney-U test).

AMR constriction time relative to Chs2p-GFP neck arrival was next examined in cells expressing *CHS2-GFP MYO1-REDSTAR* and treated with caspofungin or nikkomycin-Z (Fig 11A). In *end3*Δ mutant cells, the time taken for complete Myo1p-Redstar constriction relative to Chs2p-GFP neck arrival was 5.07 ± 0.14 min (n = 92) (Fig 11B and 11C). The time taken for complete AMR constriction in *end3*Δ cells treated with caspofungin [5.66 ± 0.19min (n = 61), p-value < 0.05] and nikkomycin-Z [6.07 ± 0.19min (n = 55), p-value<0.001] was significantly longer as compared to untreated *end3*Δ cells (Fig 11B and 11C).

Taken together, the data suggest that spindle breakage in the *end3*Δ mutant cells might be due to excessive accumulation of chitin synthase II, chitin synthase III, and glucan synthase activities at the division site during cytokinesis leading to a premature initiation and faster AMR constriction dynamics. This consequently resulted in cytokinesis in the presence of intact mitotic spindles.

### Mitotic spindle breakage in endocytosis mutants contributes to failure in spindle re-establishment in progeny cells

**Fig 12:**
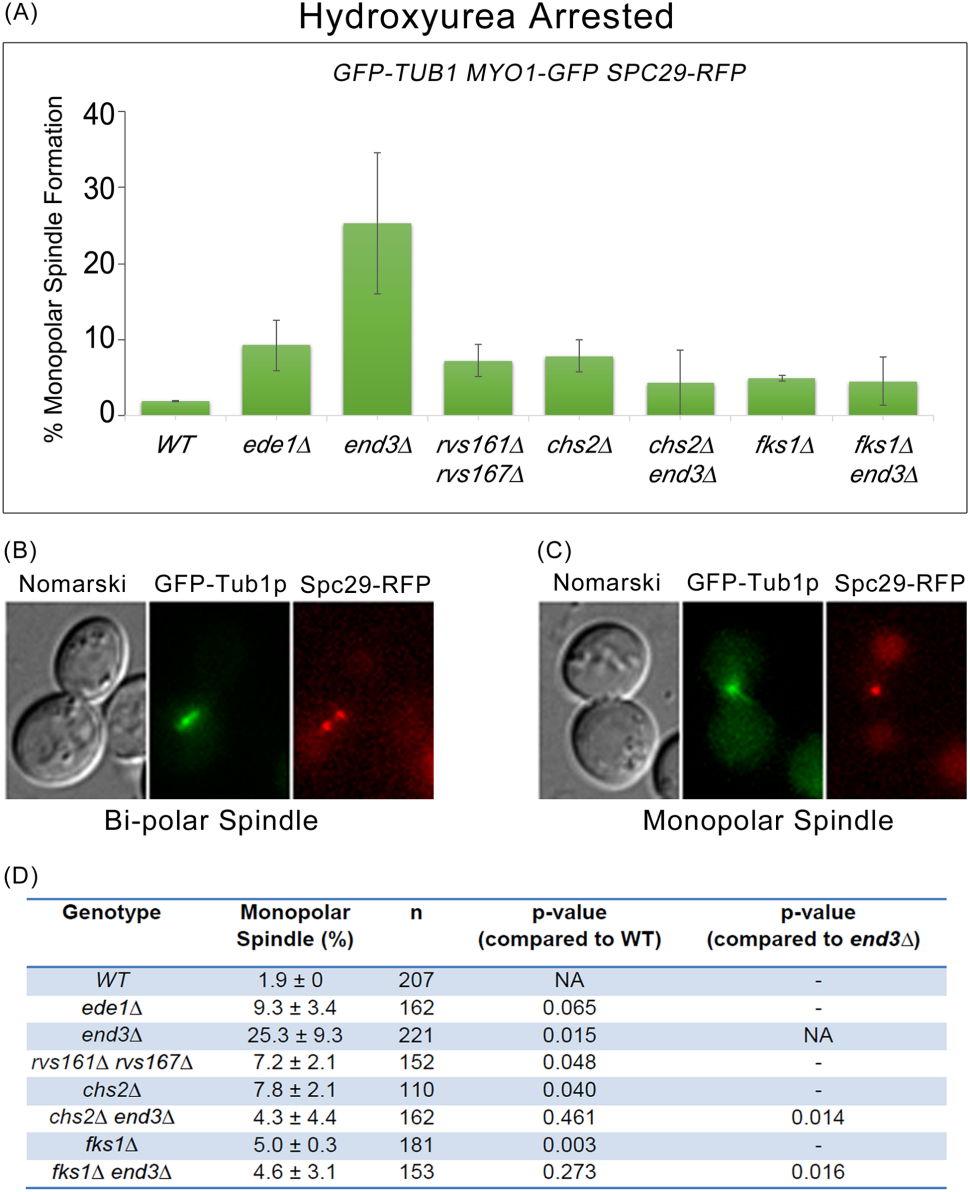
Mitotic spindle breakage in endocytosis mutants contributes to failure in spindle re-establishment in progeny cells. *GFP-TUB1 MYO1-GFP SPC29-RFP* cells harbouring deletions of key endocytic components were cultured in YPD for 2 hours at 32°C. Hydroxyurea was added to final concentration of 0.2M. Cells were arrested for 5.5 hours at 32°C and subjected to microscopy analysis. **(A)** Graph showing percentage of monopolar spindle formation in endocytosis mutants. Error bars represent SD. [**(B)** and **(C)**] Images were captured with 9x0.5μm z-planes for GFP-Tub1p Myo1p-GFP and Spc29p- RFP. The images shown were maximum projection of 9 z-planes. **(D)** Table showing the percentage monopolar spindle formation and p-values for endocytosis mutants (Student-t test).

Given that endocytosis mutants are viable in the presence of the broken spindle phenotype, we further examined the consequences of broken spindles during mitotic exit. We assessed the spindle morphology of wild-type and endocytosis mutants harbouring *GFP-TUB1 MYO1-GFP SPC29-RFP*. As a marker for SPBs, we used Spc29p, an inner plague component of the SPB [48]. Cells were cycled at 32°C for 2 hours to induce spindle breakage in endocytosis mutants, after which they were arrested using hydroxyurea (HU). Typically wild-type cells would arrest in S-phase with a short bi-polar spindle [49]. Indeed, we noted that 98.1 ± 0.05% (n = 207) of wild type cells displayed a short bipolar spindle phenotype (Fig 12A and 12B). In contrast, the endocytosis mutants, *end3*Δ and *rvs161*Δ *rvs167*Δ showed a significantly higher incidence of monopolar spindle formation as compared to wild-type cells (p-value<0.05, Fig 12A and 12C). The *end3*Δ mutant showed the highest percentage of monopolar spindle (25.3 ± 9.3%, n = 221), followed by *rvs161*Δ *rvs167*Δ (7.2 ± 2.1%, n = 152) (Fig 12D). There was no significant difference in monopolar spindle formation between wild-type and *ede1*Δ mutant cells, due to the low incidence of spindle breakage in *ede1*Δ mutant cells (Fig 12).

**Fig 13:**
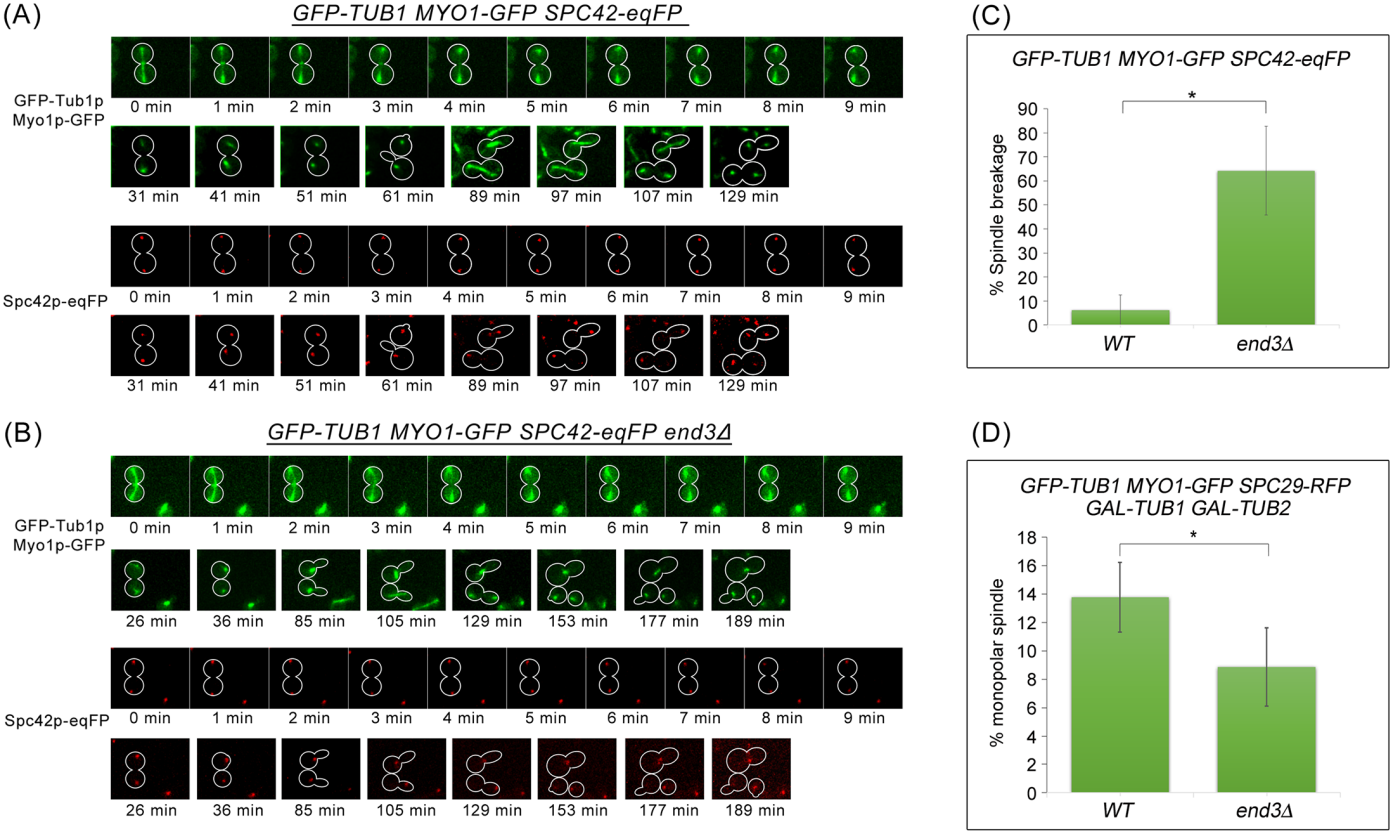
Monopolar spindles observed in *end3*Δ cells are not due SPB duplication defect. **(A)** Wild Type and **(B)** *end3*Δ cells harbouring *GFP-TUB1 MYO1-GFP SPC42-eqFP* respectively were synchronised in metaphase and then shifted up to 32°C for 30min. After release, cells were mounted on SC/Glu agar pad and examined with time-lapsed microscopy. **(C)** Graph shows percentage of spindle breakage in WT and *end3*Δ respectively (WT = 37, *end3*Δ = 38). Error bars represent SD. **(D)** WT and *end3*Δ cells harbouring *GFP-TUB1 MYO1-GFP SPC29-RFP* respectively were synchronised in metaphase and then shifted up to 32°C for 30min. After release for 60min, cells were spun into YP/Raff with 2% Gal or 2% Glu containing 0.2M HU for 5 hours. Graph showing percentage of monopolar spindles in WT and *end3*Δ cells (Student t-test, p<0.05). Error bars represent SD.

Next, we determined if the monopolar spindle formation in *end3*Δ was due to a SPB duplication failure or a defect in spindle elongation. Wild-type and *end3*Δ strains expressing *GFP-TUB1 MYO1-GFP SPC42-eqFP* were released from Noc and time-lapse imaging performed as described in Fig 7, but with an extended duration of imaging until the progeny cells underwent a subsequent round of mitosis. In wild-type cells, all progeny cells completed spindle elongation successfully in the new round of mitosis (n = 37) (Fig 13A). However, 25 out of 38 of the *end3*Δ cells had broken spindles. Of these cells with broken spindles, all of their progeny cells were able to form short bipolar spindles (Fig 13B). However, 18% of the 50 progeny cells failed to elongate their defective spindles (105min-189min; Fig 13B). Nonetheless, the data indicate that the monopolar spindles arising from spindle breakage in the endocytosis mutants are not likely due to SPB duplication failure but rather a problem with spindle elongation.

We next tested if the monopolar spindles in the endocytosis mutants can be rescued by deletion of *chs2*Δ or *fks1*Δ. However, since *chs2*Δ is inviable in the W303 genetic background yeast strain, *CHS2* was controlled under inducible galactose promoter to maintain the viability of *chs2*Δ cells. *GAL-CHS2-13MYC chs2*Δ *GFP-TUB1 MYO1-GFP SPC29-RFP* cells were grown overnight in YP/Raff medium containing 0.1% galactose. Consistent with the results from the spindle breakage rescue, there was a significant decrease in the percentage of monopolar spindle formation in *chs2*Δ *end3*Δ (4.3 ± 4.4%, n = 162, p-value <0.05) and *fks1*Δ *end3*Δ cells (4.6 ± 3.1%, n = 153, p-value <0.05) (Fig 12A and 12D). Monopolar spindle formation in *sla2*Δ, *chs3*Δ and *chs3*Δ *end3*Δ cells was not examined due to the compromised viability of these mutants in HU (S6 Fig). Furthermore, previous studies demonstrated that *chs3*Δ is synthetic lethal with endocytic components, Rvs161p, Rvs167p, and Vrp1p [50, 51]. The compromised viability of *chs3*Δ *end3*Δ double mutant cells was likely due to prolonged arrest in HU at 32°C.

The evidence indicates that spindle breakage in the endocytosis mutants leads to failure in re-establishment of spindles in G1-S phase transition. The breakage of the mitotic spindle by the AMR has been previously shown to contribute to monopolar spindle formation, presumably due to an insufficient pool of tubulin for the re-establishment of spindle in progeny cells [52]. We explored this possibility by over-expressing *TUB1* and *TUB2* in the *GFP-TUB1 MYO1-GFP SPC29-RFP end3*Δ cells. From the data, there was a low albeit statistically significant reduction in the occurrence of monopolar spindles upon galactose-induction of *TUB1* and *TUB2* (Fig 13D). This observation points to the possibility that untimely breakage of mitotic spindles could lead to a defect in generating assembly-competent tubulin in a sub-population of the progeny cells.

## Discussion

Lessons from fission yeast ‘cut’ mutants (reviewed in [53]) indicate that after cells have committed to septation, any delay or halting of the process is unlikely even if late mitotic events are compromised (point of no return). Consistent with this notion, budding yeast that exhibit defects in spindle disassembly such as *kip3*Δ mutant cells continue to undergo cytokinesis despite the presence of an intact spindle or a dicentric chromosome [54], suggesting that mitotic arrest is not an option upon initiation of septation due to the absence of a post-anaphase surveillance system in budding yeast. This raises the question of how cells regulate the septation process to ensure that cytokinesis invariably occurs after spindle disassembly.

Such a mechanism is especially pertinent for the coordination distinct processes to generate viable progeny, as cytokinetic enzymes involved in septation such as Chs2p, Chs3p, and Fks1p localize to the neck prior to spindle disassembly (Fig 1A, 1C and 1D). Particularly, given that Chs2p is the main driver of AMR constriction [6, 7], the accumulation of Chs2p at the neck could in principle lead to premature execution of cytokinesis that causes mitotic spindle breakage. Moreover, it has been shown that the regulators of Chs2p activity including Inn1p and Cyk3p are also localized to the neck during mitotic exit [10, 55–57], implies that the Chs2p is active at the neck in the presence of low mitotic CDK activity. However, typically in wild-type cells, 95% of cells that have exited from mitosis were able to disassemble spindles successfully prior to septation and AMR constriction ([21] and our data).

Some hints of how cells might coordinate cytokinesis while maintaining spindle integrity during mitotic exit could be seen from the correlation between higher levels of Chs2p, premature localization of Chs3p and Fks1p at the neck during mitotic exit, and a drastic increase in mitotic spindle breakage in endocytosis mutants (Figs 2 and 4). Moreover, the spindle breakage phenotype was rescued when either single deletions of the cytokinesis enzymes (Fig 5) or inhibitors of the enzymes were added to *end3*Δ cells (Fig 10). These data support the notion that a defect in endocytosis led to increased septation that consequently promoted AMR constriction, prematurely shearing the mitotic spindle.

Interestingly, despite the presence of the skewed distribution of the spindle-halves (Fig 6) and asymmetrical localisation of spindle midzone as marked by Ase1p-GFP on the broken spindles (Fig 7) in the endocytosis mutants, septation and AMR constriction were not inhibited. This might appear inconsistent with the reports showing that midzone mutants triggered the NoCut checkpoint that functions to delay AMR contraction and abscission [58, 59]. However, it should be noted that in the midzone mutants examined previously [58, 59], the loss-of-function of the midzone components led to unstable inter-polar microtubules that caused instability and collapse of the mitotic spindles. With defective spindles, abnormal chromosomes segregation ensued and lagging chromosomes occurred as a consequence. It was proposed that the trigger for the NoCut checkpoint that delayed AMR constriction and abscission was in fact the presence of lagging chromosomes at the midzone [58, 59]. In the endocytosis mutants, however, presumably the mitotic spindles were functional and chromosomes segregated normally. Therefore, in the endocytosis mutants, the NoCut checkpoint was not activated and AMR constriction occurred due to the accumulated activities of the cytokinetic enzymes despite the presence of intact mitotic spindles. This supports the notion that a post-anaphase surveillance system in budding yeast might not exist once chromosomes have cleared the midzone.

The findings that AMR constriction can occur in endocytosis mutants in the presence of intact mitotic spindle is significant, as cells with severed spindles could possibly enter the subsequent round of cell division to form monopolar spindles even though the SPB duplication appeared normal (Fig 13). Moreover, our data showing a marginally significant rescue of the monopolar phenotype by the overexpression of *TUB1* and *TUB2* implicates spindle breakage in causing a deficiency in the intracellular pool of assembly-competent tubulin available for progeny cells during subsequent mitoses as previously suggested [52]. However, while a significant number of *end3*Δ cells exhibited broken spindles (72.2 ± 3.3%, n = 119), only (25.3 ± 9.3%, n = 221) had monopolar spindles (Figs 5 and 12). The figures are similar to the *kip3*Δ mutant and would suggest that the cells are fairly robust in terms of recovery from spindle breakage. Indeed, the synergistic effects on spindle breakage in the *kip3*Δ *end3*Δ double mutant point to the possibility that spindle assembly is a complex process that perhaps relies on several redundant pathways to ensure the formation of functional mitotic spindles in progeny cells embarking on subsequent rounds of cell division.

The mechanisms underlying tight coordination of the final events of mitosis has been the subject of various studies, with a substantial focus on the mitotic kinase activity as the main determinant. Work from other labs have demonstrated that a decline in mitotic CDK1 activity and the release of the late mitotic phosphatase Cdc14p could lead to the reversal of the action of the mitotic CDK1 activity on several substrates related to these processes. Mitotic CDK1 activity therefore appears to play a key part in the coordination of spindle disassembly, septation and AMR constriction. For instance, spindle disassembly depends upon the APC^Cdh1^ complex that is activated upon reduction in mitotic kinase activity. APC^Cdh1^is needed for the destruction of midzone proteins Cin8p and Ase1p that function early in mitosis for spindle elongation [20, 60]. Septation is also contingent upon the export of Chs2p from the RER when mitotic CDK1 activity is sufficiently reduced and Cdc14p released [8, 9, 26]. Furthermore, AMR constriction requires low mitotic kinase, in part due to the presence of Chs2p and in part due to the localization of MEN components to the neck [61], though the functions of the MEN components at the neck remain unknown.

However, our data showing that premature AMR constriction could occur during the earlier phase of mitosis by the mere presence of elevated levels of cytokinesis enzymes highlights the fact that the level of mitotic CDK1 activity is not directly/solely responsible for coordinating spindle disassembly and cytokinesis. Moreover, the fact that the cytokinetic enzymes localise to the neck during mitotic exit prior to spindle disassembly further argues against the case for the drop in mitotic kinase activity as being a key factor coordinating these mitotic events. Rather, other processes such as endocytosis that might appear not to be directly regulated by mitotic activity could also contribute to the timeliness of cell cycle events. This is important as the trigger for endocytosis appears to be due to the presence of cargo. For instance, as shown from a previous study, the presence of endocytic cargo at the membrane could trigger the endocytic process leading to the internalisation from the plasma membrane [31]. In the case of the cytokinetic enzymes, this places a negative feedback loop that regulates the levels of the enzymes that could contribute to septation and AMR constriction without compromising spindle integrity.

**Fig 14:**
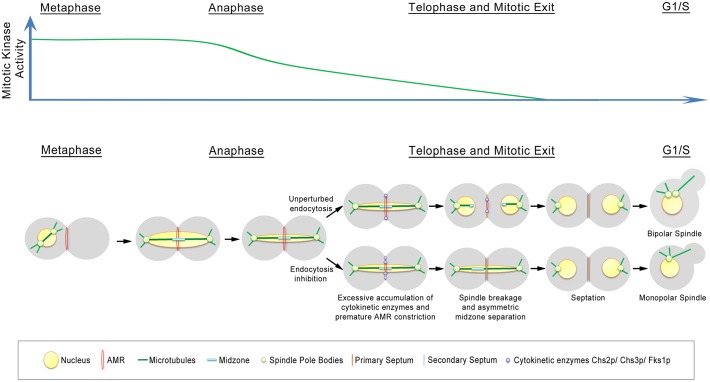
Proposed model. Coordination of mitotic spindle disassembly, endocytosis of cytokinetic enzymes endocytosis and cytokinesis during mitotic exit.

Our model places the retrieval of endocytosis proteins within the normal regulation of septation, AMR constriction and spindle assembly (Fig 14). As such, while it has been previously shown that endocytosis of Chs2p and Chs3p occurred during late mitosis to remove them from the neck subsequent to septation [6, 9, 24], our data suggest that the retrieval process is in fact active all throughout mitotic exit (Fig 3). This suggestion is borne out by our data showing that endocytosis components are found at the neck very soon after Chsp2-mCherry appears at the neck (S2 Fig). In *CHS2-mCHERRY ABP1-GFP* cells, the Chs2p-mCherry preceded Abp1p-GFP in the neck localisation by 1.9 ± 1.25min (S2 Fig), though Chs2p-mCherry and Abp1p-mCherry can be observed 2.06 ± 0.80min and 1.90 ± 0.99min respectively before spindle disassembly when compared to GFP-Tub1p. There is some variablity in the timings when examining Chs2p-mCherry and Abp1p-mCherry across different strains but it is important to note that the differences do not account for the heightened levels of Chs2p-GFP accumulation in the endocytosis mutants (Fig 2). It is clear that both Chs2p and Abp1p arrived at the neck prior to spindle disassembly (Fig 1A and 1E). More crucially, at the bud necks of wild-type cells, cytokinetic enzymes such as Chs2p-GFP neck levels do not accumulate to levels that are found in endocytosis mutants at any point in time (Fig 2).

We propose that during mitotic exit when cytokinetic enzymes such as Chs2p, Chs3p and Fks1p are trafficked to the plasma membrane at the neck and are internalised soon after their arrival. Presumably, the rate of endocytosis of these enzymes is slower than the forward trafficking process that eventually leads to the accumulation of these enzymes at the neck. Presently, it is unclear how the differential rates of forward trafficking and endocytosis are maintained, though the distinct rates might be inherent properties of the two pathways and the net levels of the enzymes at the neck are due to stochastic accumulation. Alternatively, it is also possible that the orientation of the actin cytoskeleton during mitotic exit towards the neck favours forward trafficking relative to endocytosis. This results in a net accumulation of cytokinetic enzymes at the neck that is sufficient to trigger AMR constriction, though the levels required for AMR constriction are reached only after spindle disassembly due to the constitutive endocytosis of the cytokinetic enzymes.

At one level, our data implicates endocytosis in the maintenance of spindle integrity, a novel role not previously identified. At another level, our data demonstrates that the interplay between cell cycle-dependent events, such as directional trafficking of cytokinetic enzymes and spindle disassembly, and cell cycle-independent events, such as endocytosis of cytokinetic enzymes, could contribute towards the timely execution of late mitotic events occurring during cell division. Taken together, our data further highlight the complexity underlying the coordination of septation, AMR constriction, and spindle disassembly during late mitosis.

## Materials and Methods

### Yeast strains and culture reagents

Yeast strains used in this study are listed in S1 and S2 tables. A combination of standard molecular biology and molecular genetics techniques such as tetrad dissection, PCR-based tagging and deletion of endogenous genes [62] were used to construct plasmids and strains. The plasmids for the GFP and mCherry cassettes were obtained from European *Saccharomyces cerevisiae* Archive for Functional analysis (Euroscarf) and Yeast Resource Centre (YRC). Tdtomato (Addgene plasmid 35193) [63], 3mGFP (Addgene plasmid # 25449) [64], and pHIS3p:mRuby2-Tub1+3'UTR::LEU2 (Addgene plasmid # 50645) [65] constructs were gifts from Robert Singer, Benjamin Glick and Wei-Lih Lee respectively. Constructs for auxin inducible degron (AID) system such as Mini-AID and ADH1-yeOSTIR1 were provided by the National BioResource Project (NBRP) of the Ministry of Education, Culture, Sports, Science and Technology (MEXT), Japan. Further information regarding the strains and plasmids construction will be provided upon request. Yeast strains were routinely grown in yeast extract peptone (YP) or selective medium supplemented with 2% dextrose (Glu) at 24°C. For galactose promoter induction, cells were grown in YP supplemented with 2% raffinose (Raff), followed by addition of galactose (Gal) to a final concentration of 0.1%.

### Cell synchronisation

For cells synchronisation, exponential-phase cells were diluted to 10^7^ cells/ml in growth medium at 24°C. For a typical Noc arrest, cells were arrested with 7.5 μg/ml Noc (US Biological) for 2h, followed by addition of 7.5 μg/ml for another 2h at 24°C. For endocytosis deletion mutants, cells were shifted to 32°C for another 0.5h after 4 hours Noc arrest. The drug was washed off by centrifugation of the cells. For S-phase arrest, cells were grown at 32°C for 2 h, followed by addition of hydroxyurea (US biological) to a final concentration of 0.2M. Cells were then incubated for another 5.5 h at 32°C and harvested for fluorescent microscopy analysis.

### Western blot analysis

Samples were collected from the respective time points stated. Protein lysates were prepared using TCA precipitation method as described previously [8]. Anti-Cdc28 (1:1000 dilution), anti-Clb2 (1:10000 dilution) (Santa Cruz, CA), anti-Chs2 (1:500) (GeneScript), anti-AID (1:5000 dilution) (MBL, Japan) and anti-Pgk1 (1:100000 dilution) (Invitrogen) were used to probe the respective proteins. The Clarity enhanced chemiluminescence kit (Bio-rad) was used according to the manufacturer’s recommendations.

### Transmission electron microscopy

Cells for TEM analysis were grown in YP supplemented with 2% glucose at 32°C to mid-log phase, fixed with 3% glutaraldehyde, then processed for observation as described in [54].

### Wide-field time-point fluorescence microscopy and time-lapse microscopy

Samples for fluorescence microscopy were taken at time points indicated in the relevant sections. Cells harbouring fluorescence protein fusions harvested by centrifugation and washed once with dH_2_O. Samples were observed directly without fixation using an IX81 wide-field fluorescence microscope (Olympus) with 60x NA 1.4 oil lens, and 1.5x optivar. Filter sets for fluorescence microscopy were purchased from Omega and Semrock, and images were capture using a CCD camera (CoolSnap HQ, Photometrics). Images acquisition was controlled by Metamorph software (Molecular Devices). Typically, the exposure time for the acquisition of the images was 0.25s for GFP and 0.30s for RFP per plane. Nine-optical Z-sections at 0.5μm intervals were obtained for each time point. Images shown were either maximal projection of the Z-stacks or images taken at a single plane, as indicated in the relevant sections. For spindle collapsed analysis, 3D reconstruction of Z-stack captured was performed using Metamorph to ensure the monopolar spindles observed were not bi-polar spindles that positioned perpendicular to the slide. At least 50 cells were counted for each time-point from 3 independent experiments.

For time-lapsed microscopy, cells released from Noc arrest were resuspended in complete synthetic medium containing glucose and mounted onto 5% agarose pad on slides. Time-lapsed images were captured as described in pervious section. Zero-drift compensation, ZDC (Olympus) was used for focal drift correction throughout the time-lapsed microscopy. For time-lapsed microscopy at 32°C, stage top incubator was used to control the temperature. ImageJ (National Institutes of Health, Bethesda, MA) and Photoshop (Adobe, San Jose, CA) were used for the production of the montages and figures.

### Spinning disk confocal time-lapsed microscopy

Cells were treated as described in the wide-field time-lapsed microscopy. Spinning disk images were captured using Olympus IX81-ZDC microscope, with a 60x NA 1.4 oil lens. Sapphire LP 488nm and 561nm solid-state lasers (Coherent) were used for the samples excitations. Filter sets for fluorescence microscopy were purchased from Omega and Semrock, and images were capture using the Photometrics 512EM-CCD attached behind the Yokogawa CSU22 connected to the microscope. GFP and RFP images were captured simultaneously using via a Dual-View image splitter (Optical Insights). Images acquisition was controlled by Metamorph software (Molecular Devices). Typically, the exposure time for the acquisition of the images was 0.2–0.35s per plane. 9-optical Z-sections at 0.5μm were obtained for each time point. Images shown were either maximal projection of the Z-stacks. For time-lapsed microscopy at 32°C, stage top incubator was used to control the temperature. ImageJ (National Institutes of Health, Bethesda, MA) and Photoshop (Adobe, San Jose, CA) were used for the production of the montages and figures.

## Supporting Information

S1 Fig Functional assay of cytokinetic enzymes tagged with green fluorescent proteins. Serial diluted cultures were spotted on YPD, YPD containing 1mM 1-Naphthalenacetic acid (NAA) and incubated at 24°C.
(TIF)

S2 Fig Chs2p-mCherry neck localization precedes neck accumulation of key CME components-GFP. *CHS2-mCHERRY* **(A)** *EDE1-GFP* (n = 63), **(B)** *SLA2-GFP* (n = 43), **(C)** *LAS17-GFP* (n = 44), **(D)** *SLA1-GFP* (n = 30), **(E)** *ABP1-GFP* (n = 61), **(F)** *RVS167-GFP* (n = 52) cells were synchronised in metaphase with Noc. After release, cells were mounted on SC/Glu agar pad and examined with time-lapsed microscopy.
(TIF)

S3 Fig Ultrastructure analysis of endocytic deletion mutants. **(A)** Wild Type, **(B)** *end3*Δ, and **(C)** *sla2*Δ cells for TEM analysis were grown in YP supplemented with 2% glucose at 32°C to mid-log phase, fixed with 3% glutaraldehyde, then processed for observation.
(TIF)

S4 Fig Key CME component deletion mutant does not display defect in mitotic exit. **(A)** Yeast strains harbouring the endocytic component deletions were synchronised in metaphase. After 4 hours, cells were shifted to 32°C for 30min. Cells were then released from metaphase into pre-warmed 32°C YPD. Western blot analysis of Clb2p, Cdc28p and Pgk1p levels are shown to demonstrate equivalent mitotic exit during release from Noc. **(B)** Graph shows the Clb2p signals normalized against loading control Pgk1.
(TIF)

S5 Fig Characterization of *CHS2-1xMini-AID* mutant cell. **(A)** Serial diluted cultures of *wild type*, *end3*Δ, *chs2-1xMini-AID* and *chs2-1xMini-AID end3*Δ harbouring *ADH1-yeOSTIR1* were spotted on YPD, YPD containing 0.25mM IAA or YPD containing 0.5mM IAA and incubated at 24°C. **(B)** Cells from YPD, YPD containing 0.25mM IAA or YPD+0.5mM IAA plate were subjected to microscopy analysis.
(TIF)

S6 Fig Hydroxyurea sensitivity test on endocytosis mutants. Serial diluted cultures of were spotted on YPD, YP/Raff/Gal, YPD or YP/Raff/Gal containing 25mM, 50mM, 100mM, and 200mM HU respectively, and incubated at 24°C.
(TIF)

S1 Table Yeast strains used in this study. (DOCX)

S2 Table Yeast strains used in supplemental data. (DOCX)
