# Supplementary figures and images for: Timely Endocytosis of Cytokinetic Enzymes Prevents Premature Spindle Breakage during Mitotic Exit
Source: PLoS Genet. 2016 Jul 22;12(7):e1006195. doi: 10.1371/journal.pgen.1006195 (PMC4957831; doi:10.1371/journal.pgen.1006195)

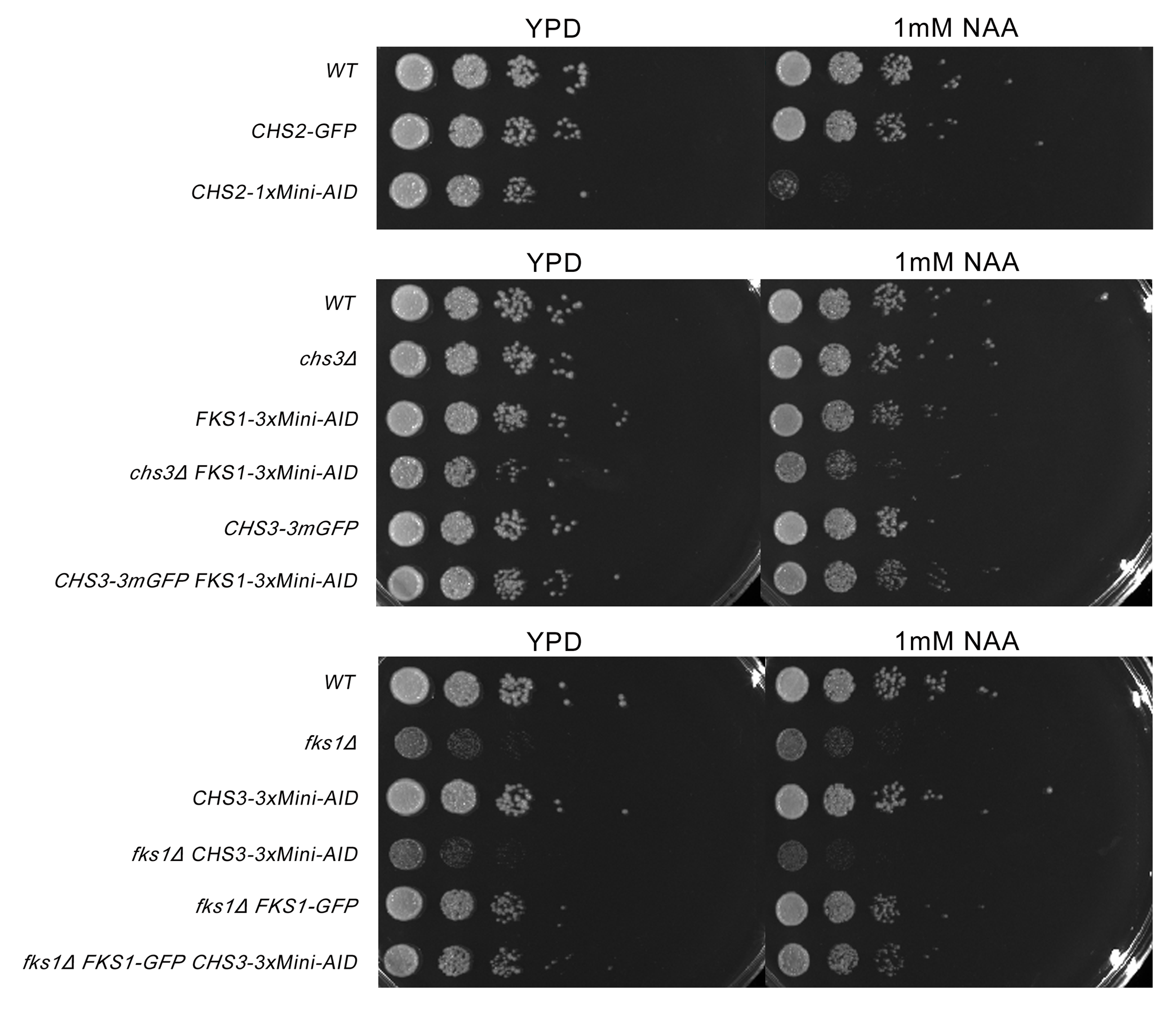

Supplement: S1 Fig — Serial diluted cultures were spotted on YPD, YPD containing 1mM 1-Naphthalenacetic acid (NAA) and incubated at 24°C. (TIF) [file pgen.1006195.s001.tif]

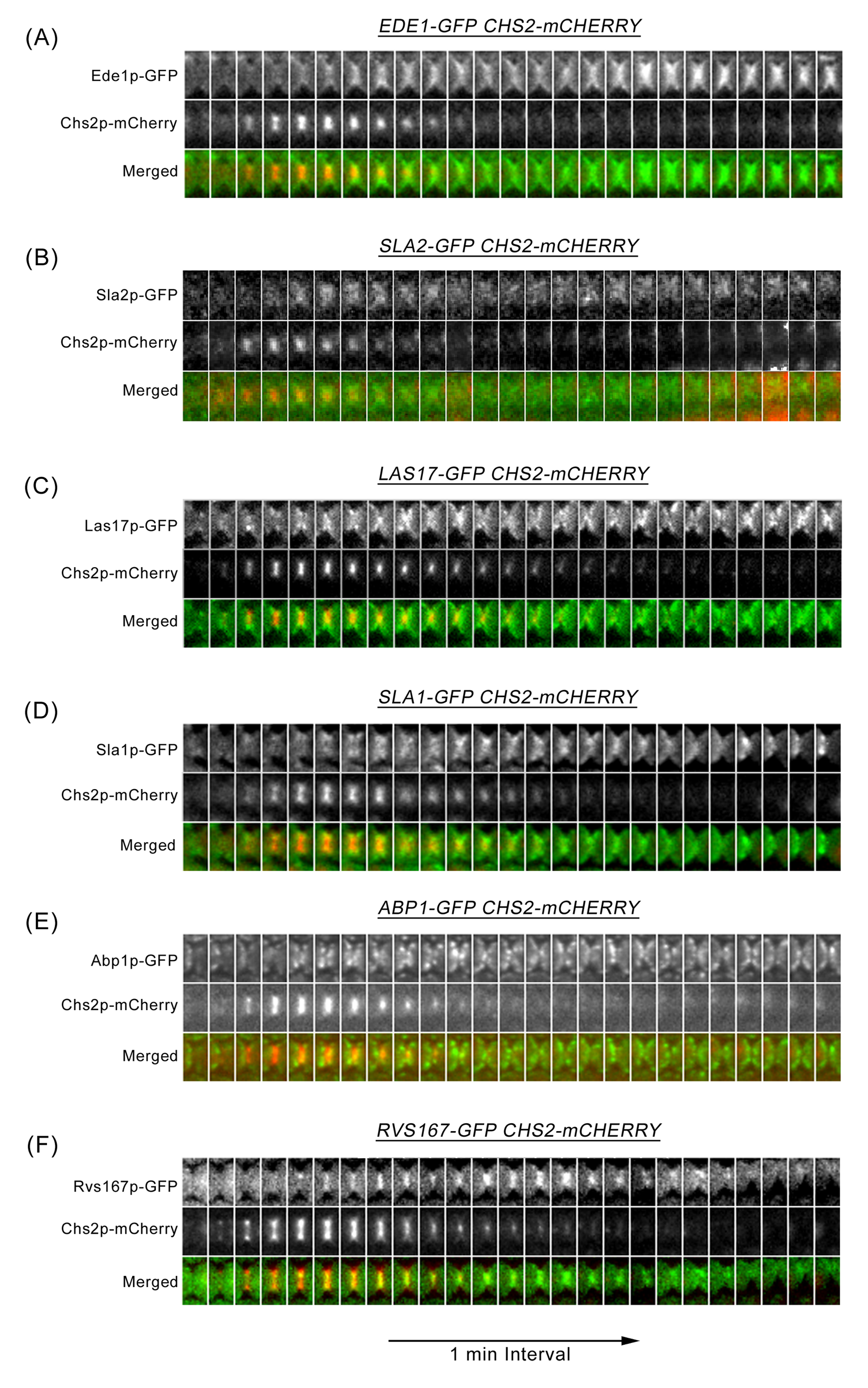

Supplement: S2 Fig — CHS2-mCHERRY (A) EDE1-GFP (n = 63), (B) SLA2-GFP (n = 43), (C) LAS17-GFP (n = 44), (D) SLA1-GFP (n = 30), (E) ABP1-GFP (n = 61), (F) RVS167-GFP (n = 52) cells were synchronised in metaphase with Noc. After release, cells were mounted on SC/Glu agar pad and examined with time-lapsed microscopy. (TIF) [file pgen.1006195.s002.tif]

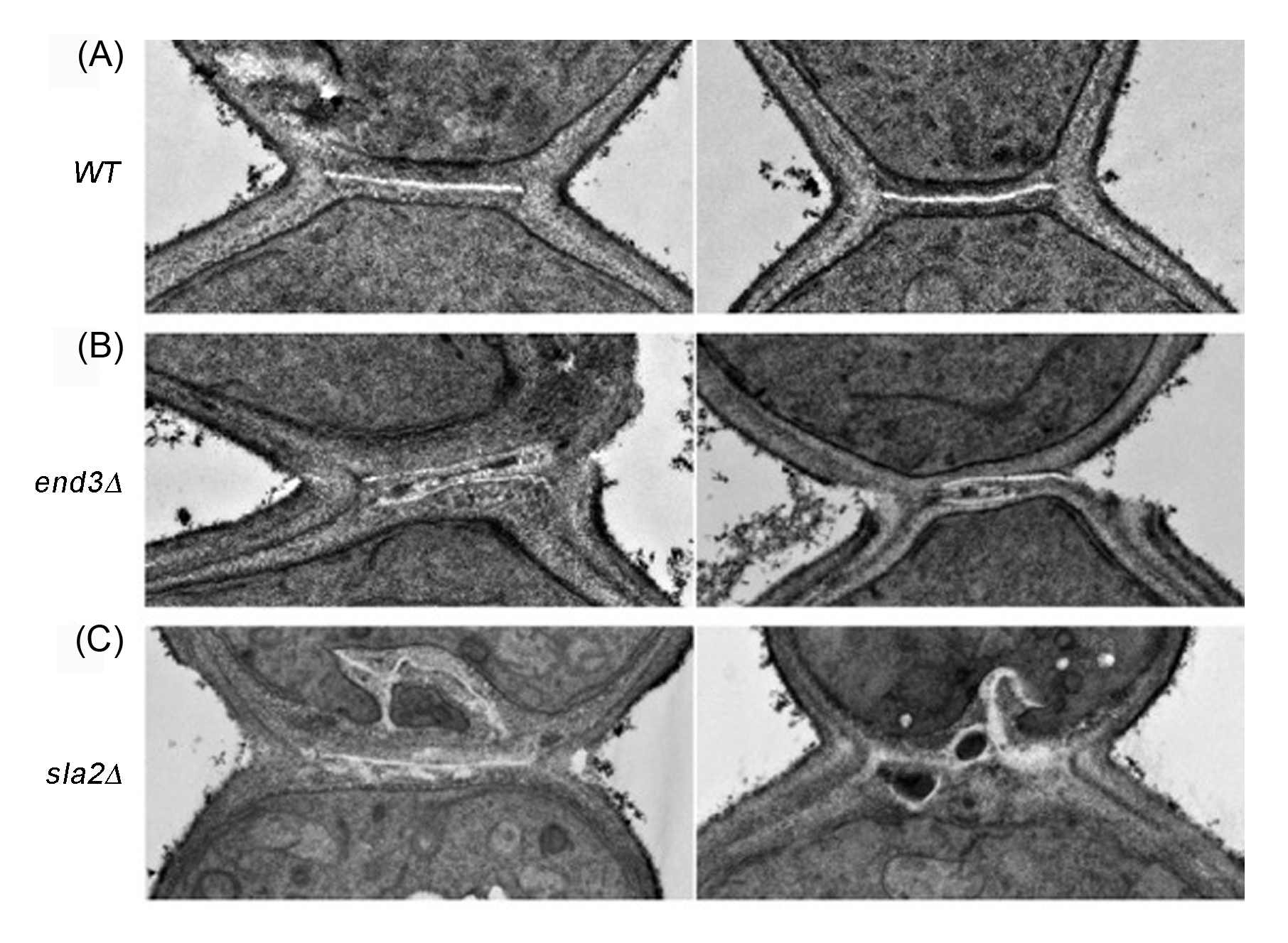

Supplement: S3 Fig — (A) Wild Type, (B) end3Δ, and (C) sla2Δ cells for TEM analysis were grown in YP supplemented with 2% glucose at 32°C to mid-log phase, fixed with 3% glutaraldehyde, then processed for observation. (TIF) [file pgen.1006195.s003.tif]

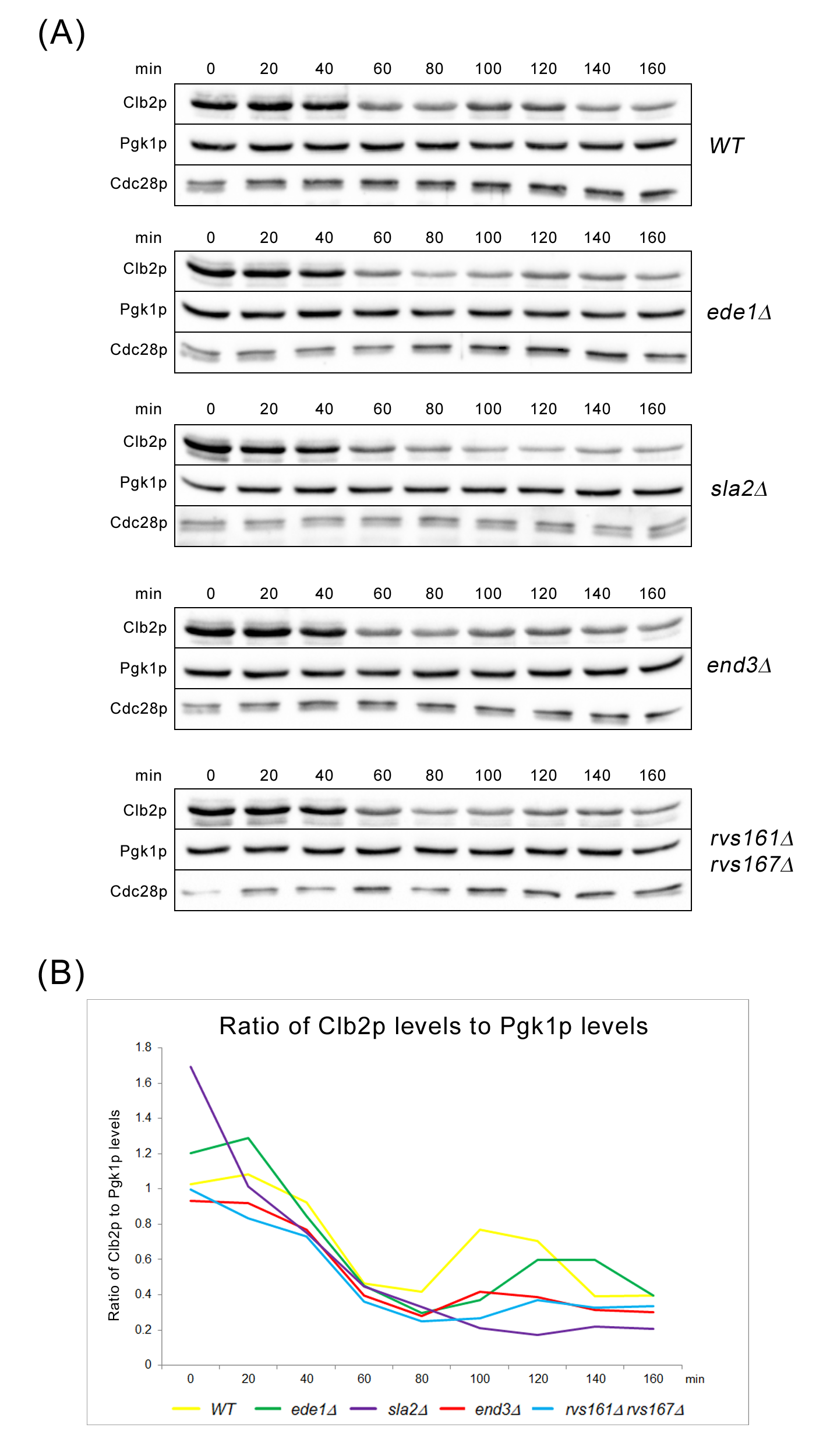

Supplement: S4 Fig — (A) Yeast strains harbouring the endocytic component deletions were synchronised in metaphase. After 4 hours, cells were shifted to 32°C for 30min. Cells were then released from metaphase into pre-warmed 32°C YPD. Western blot analysis of Clb2p, Cdc28p and Pgk1p levels are shown to demonstrate equivalent mitotic exit during release from Noc. (B) Graph shows the Clb2p signals normalized against loading control Pgk1. (TIF) [file pgen.1006195.s004.tif]

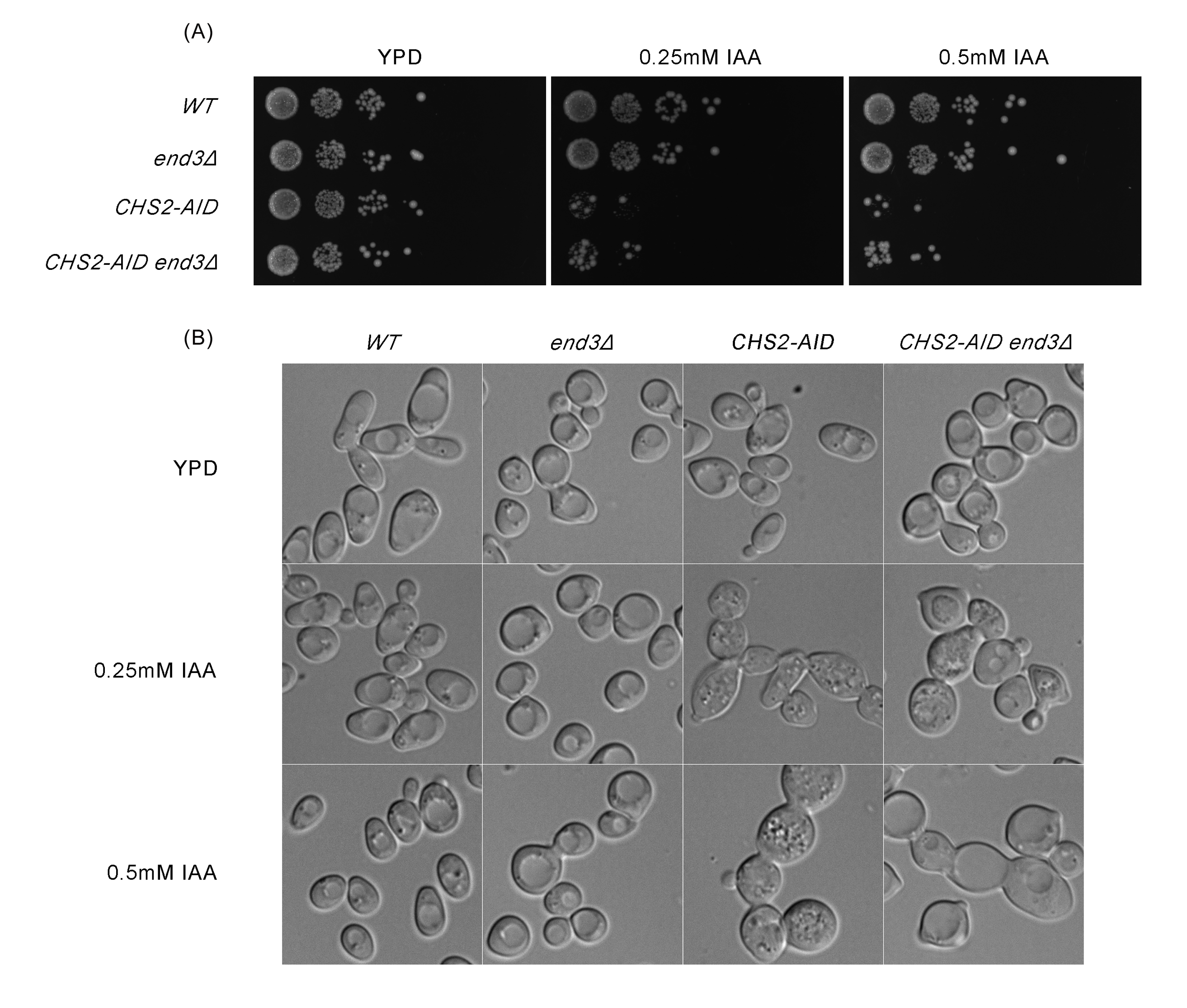

Supplement: S5 Fig — (A) Serial diluted cultures of wild type, end3Δ, chs2-1xMini-AID and chs2-1xMini-AID end3Δ harbouring ADH1-yeOSTIR1 were spotted on YPD, YPD containing 0.25mM IAA or YPD containing 0.5mM IAA and incubated at 24°C. (B) Cells from YPD, YPD containing 0.25mM IAA or YPD+0.5mM IAA plate were subjected to microscopy analysis. (TIF) [file pgen.1006195.s005.tif]

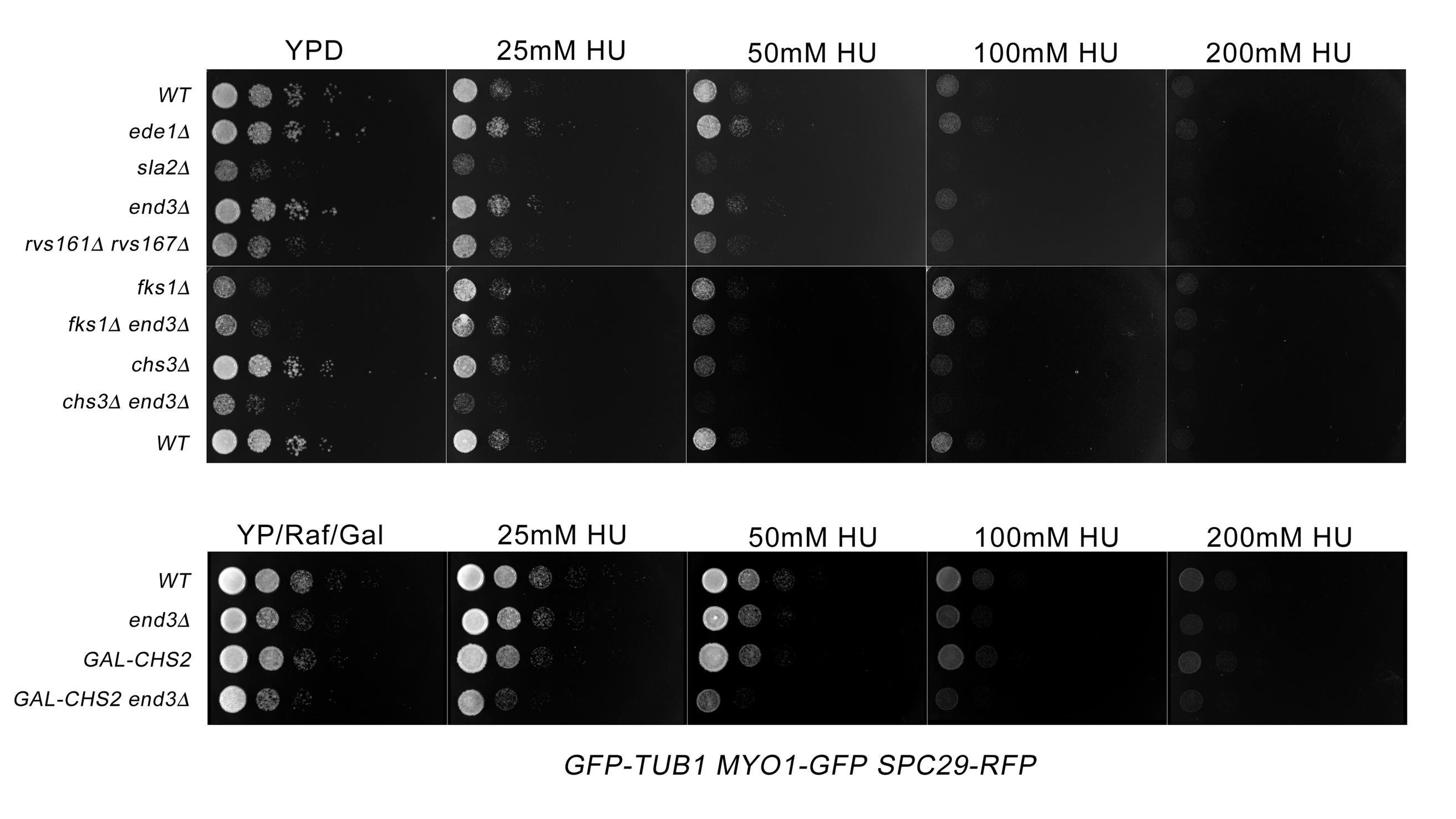

Supplement: S6 Fig — Serial diluted cultures of were spotted on YPD, YP/Raff/Gal, YPD or YP/Raff/Gal containing 25mM, 50mM, 100mM, and 200mM HU respectively, and incubated at 24°C. (TIF) [file pgen.1006195.s006.tif]
