# Supplementary material for: Timely Endocytosis of Cytokinetic Enzymes Prevents Premature Spindle Breakage during Mitotic Exit
Source: PLoS Genet. 2016 Jul 22;12(7):e1006195. doi: 10.1371/journal.pgen.1006195 (PMC4957831; doi:10.1371/journal.pgen.1006195)
Supplement: S1 Table — (DOCX) [file pgen.1006195.s007.docx]

**S1 Table. Yeast Strains used in this study.**

| Name | Genotype | Source | |
| --- | --- | --- | --- |
| US1363 | *MAT a leu2-3,112 trp1-1 ura3-1 can1-100 ade2-1 his3-11,15 bar1Δ* | | Uttam Surana |
| KR3212 | *MATa SPC42-RFP-KanMX6 GFP-TUB1-URA3::TUB1 ura3-1 his3-11,15 leu2-3,112* | | Orna Cohen-Fix |
| Y1125 | *MAT a ade2 his3 lys2 trp1 ura3 fks1::HIS ade3::FKS1-GFP::TRP1 fks2::LYS* | | Yu Jiang |
| FM2705 | *MAT a MYO1-GFP::KAN GFP-TUB1::URA CHS2-mCHERRY::hphNT1* | This Study | |
| FM3561 | *MAT a CHS2-mCHERRY::hphNT1 CHS3-3mGFP::KAN* | This Study | |
| FM3426 | *MAT a fks1::HIS ade3::FKS1-GFP::TRP1 CHS2-mCHERRY* | This Study | |
| FM2933 | *MAT a MYO1-GFP::KAN GFP-TUB1::URA ABP1-mCHERRY::hphNT1* | This Study | |
| FM2412 | *MAT a CHS2-GFP::KAN ABP1-mCHERRY::hphNT1* | This Study | |
| FM2442 | *MAT a CHS2-GFP::KAN ABP1-mCHERRY::hphNT1 ede1::natNT2* | This Study | |
| FM2713 | *MAT a CHS2-GFP::KAN ABP1-mCHERRY::natNT2N sla2::hphNT1* | This Study | |
| FM2515 | *MAT a CHS2-GFP::KAN ABP1-mCHERRY::hphNT1 end3::natNT2* | This Study | |
| FM3221 | *MAT a CHS2-GFP::KAN ABP1-mCHERRY::hphNT1 rvs167::natNT2 rvs161::HIS* | This Study | |
| FM4896 | *MAT a SPC42-eqFP611::hphNT1 GAL-CHS2(6S-6A)-GFP::URA* | This Study | |
| FM4865 | *MAT a cdc15-2 GAL-CHS2(6S-to-6A)-GFP::URA SPC42-eqFP611::hphNT1* | This Study | |
| FM3376 | *MAT a fks1::HIS ade3::FKS1-GFP::TRP1 ABP1-mCHERRY::hphNT1* | This Study | |
| FM3525 | *MAT a ABP1-mCHERRY::hphNT1 CHS3-3mGFP::KAN* | This Study | |
| FM2642 | *MAT a MYO1-GFP::KAN GFP-TUB1::URA* | This Study | |
| FM3046 | *MAT a MYO1-GFP::KAN GFP-TUB1::URA ede1::natNT2* | This Study | |
| FM3260 | *MAT a MYO1-GFP::KAN GFP-TUB1::URA sla2::natNT2* | This Study | |
| FM2665 | *MAT a MYO1-GFP::KAN GFP-TUB1::URA end3::natNT2* | This Study | |
| FM3262 | *MAT a MYO1-GFP::KAN GFP-TUB1::URA rvs161::HIS rvs167::natNT2* | This Study | |
| FM2873 | *MAT α GFP-TUB1::URA MYO1-GFP::KAN chs3::KAN* | This Study | |
| FM2889 | *MAT α GFP-TUB1::URA MYO1-GFP::KAN chs3::KAN end3::natNT2* | This Study | |
| FM3191 | *MAT a MYO1-GFP::KAN GFP-TUB1::URA fks1::hphNT1* | This Study | |
| FM3198 | *MAT a MYO1-GFP::KAN GFP-TUB1::URA fks1::hphNT1 end3::natNT2* | This Study | |
| FM3314 | *MAT a MYO1-GFP::KAN GFP-TUB1::URA kip3::hphNT1* | This Study | |
| FM3340 | *MAT a MYO1-GFP::KAN GFP-TUB1::URA kip3::hphNT1 end3::natNT2* | This Study | |
| FM3471 | *MAT a MYO1-GFP::KAN GFP-TUB1::URA slk19::natNT2* | This Study | |
| FM3487 | *MAT a MYO1-GFP::KAN GFP-TUB1::URA slk19::natNT2 end3::hphNT1* | This Study | |
| FM4878 | *MAT a MYO1-tdTOMATO::loxp::KAN::loxp ASE1-GFP::TRP1 pHIS3-mRUBY2-TUB1::hphNT1* | This Study | |
| FM4896 | *MAT a MYO1-tdTOMATO::loxp::KAN::loxp ASE1-GFP::TRP1 pHIS3-mRUBY2-TUB1::hphNT1 end3::natNT2* | This Study | |
| FM3440 | *MAT a MYO1-GFP::TRP CHS2-mCHERRY::hphNT1* | This Study | |
| FM4070 | *MAT a MYO1-GFP::TRP CHS2-mCHERRY::hphNT1 ede1::natNT2* | This Study | |
| FM4073 | *MAT a MYO1-GFP::TRP CHS2-mCHERRY::hphNT1 sla2::natNT2* | This Study | |
| FM4074 | *MAT a MYO1-GFP::TRP CHS2-mCHERRY::hphNT1 end3::natNT2* | This Study | |
| FM4130 | *MAT a MYO1-GFP::TRP CHS2-mCHERRY::hphNT1 rvs161::HIS rvs167::natNT2* | This Study | |
| FM3950 | *MAT a TUB1-GFP::URA MYO1-GFP::TRP* | This Study | |
| FM4852 | *MAT a TUB1-GFP::URA MYO1-GFP::TRP lsb1::natNT2 lsb2::hphNT1* | This Study | |
| FM4525 | *MAT a MYO1-GFP::TRP GFP-TUB1::URA ADH1-yeOsTIR1::LEU* | This Study | |
| FM4703 | *MAT a MYO1-GFP::TRP GFP-TUB1::URA ADH1-yeOsTIR1::LEU end3::natNT2* | This Study | |
| FM4543 | *MAT a MYO1-GFP::TRP GFP-TUB1::URA ADH1-yeOsTIR1::LEU CHS2-1x Mini-AID::HIS* | This Study | |
| FM4549 | *MAT a MYO1-GFP::TRP GFP-TUB1::URA ADH1-yeOsTIR1::LEU CHS2-1x Mini-AID::HIS end3::natNT2* | This Study | |
| FM3106 | *MAT a CHS2-GFP::KAN MYO1-REDSTAR::natNT2* | This Study | |
| FM3111 | *MAT a CHS2-GFP::KAN MYO1-REDSTAR::natNT2 end3::hphNT1* | This Study | |
| FM2784 | *MAT a MYO1-GFP::KAN GFP-TUB1::URA SPC29-RFP::hphNT1* | This Study | |
| FM2908 | *MAT a MYO1-GFP::KAN GFP-TUB1::URA SPC29-RFP::hphNT1 ede1::natNT2* | This Study | |
| FM2817 | *MAT a MYO1-GFP::KAN GFP-TUB1::URA SPC29-RFP::hphNT1 end3::natNT2* | This Study | |
| FM3224 | *MAT a MYO1-GFP::KAN GFP-TUB1::URA SPC29-RFP::hphNT1 rvs167::natNT2 rvs161::HIS* | This Study | |
| FM3349 | *MAT a MYO1-GFP::KAN GFP-TUB1::URA SPC29-RFP::hphNT1 GAL-CHS2-13MYC::TRP chs2::HIS* | This Study | |
| FM3200 | *MAT a MYO1-GFP::KAN GFP-TUB1::URA SPC29-RFP::hphNT1 GAL-CHS2-13MYC::TRP chs2::HIS end3::natNT2* | This Study | |
| FM3285 | *MAT a MYO1-GFP::KAN GFP-TUB1::URA SPC29-RFP::hphNT1 fks1::LEU* | This Study | |
| FM3296 | *MAT a MYO1-GFP::KAN GFP-TUB1::URA SPC29-RFP::hphNT1 fks1::LEU end3::natNT2* | This Study | |
| FM4245 | *MAT a MYO1-GFP::KAN GFP-TUB1::URA SPC42-eqFP611::hphNT1* | This Study | |
| FM4805 | *MAT a MYO1-GFP::KAN GFP-TUB1::URA SPC42-eqFP611::hphNT1 end3::natNT2* | This Study | |
| FM4914 | *MAT a MYO1-GFP::KAN GFP-TUB1::URA SPC29-RFP::hphNT1 end3::natNT2 GAL-TUB1::TRP GAL-TUB2::LEU* | This Study | |

*natNT2* refers to Nourseothricin

*hphNT1* refers to Hygromycin B
