# Supplementary material for: Timely Endocytosis of Cytokinetic Enzymes Prevents Premature Spindle Breakage during Mitotic Exit
Source: PLoS Genet. 2016 Jul 22;12(7):e1006195. doi: 10.1371/journal.pgen.1006195 (PMC4957831; doi:10.1371/journal.pgen.1006195)
Supplement: S2 Table — (DOCX) [file pgen.1006195.s008.docx]

**S2 Table. Yeast Strains used in Supplemental Data**

| Name | *Genotype* | Source |
| --- | --- | --- |
| FM4613 | *MAT a ADH3-yeOsTIR1::LEU2* | This Study |
| FM4799 | *MAT a ADH3-yeOsTIR1::LEU2 CHS2-GFP::KAN* | This Study |
| FM4825 | *MAT a ADH3-yeOsTIR1::LEU2 CHS2-1xMini-AID::HIS3* | This Study |
| FM4840 | *MAT a ADH3-yeOsTIR1::LEU2 chs3::natNT2* | This Study |
| FM4816 | *MAT a ADH3-yeOsTIR1::LEU2 FKS1-3xMini-AID::HIS3* | This Study |
| FM4820 | *MAT a ADH3-yeOsTIR1::LEU2 chs3::natNT2 FKS1-3xMini-AID::HIS3* | This Study |
| FM4801 | *MAT a ADH3-yeOsTIR1::LEU2 CHS3-3mGFP::KAN* | This Study |
| FM4813 | *MAT a ADH3-yeOsTIR1::LEU2 CHS3-3mGFP::KAN FKS1-3xMini-AID::HIS3* | This Study |
| FM4802 | *MAT a ADH3-yeOsTIR1::LEU2 fks1::natNT2* | This Study |
| FM4807 | *MAT a ADH3-yeOsTIR1::LEU2 CHS3-3xMini-AID::KAN* | This Study |
| FM4823 | *MAT a ADH3-yeOsTIR1::LEU2 fks1::natNT2 CHS3-3xMini-AID::KAN* | This Study |
| FM4803 | *MAT a ADH3-yeOsTIR1::LEU2 ade3::FKS1-GFP::TRP fks1::HIS3* | This Study |
| FM4807 | *MAT a ADH3-yeOsTIR1::LEU2 ade3::FKS1-GFP::TRP fks1::HIS3 CHS3-3xMini-AID::KAN* | This Study |
| FM2416 | *MAT a EDE1-GFP::HIS CHS2-mCHERRY::hphNT1* | This Study |
| FM2534 | *MAT a CHS2-mCHERRY::hphNT1 SLA2-GFP::KAN* | This Study |
| FM2315 | *MAT a LAS17-GFP::HIS3 CHS2-mCHERRY::hphNT1* | This Study |
| FM2345 | *MAT a CHS2-mCHERRY::hphNT1 SLA1-GFP::KAN* | This Study |
| FM2420 | *MAT a ABP1-GFP::HIS CHS2-mCHERRY::hphNT1* | This Study |
| FM2347 | *MAT a RVS167-GFP::HIS CHS2-mCHERRY::hphNT1* | This Study |
| FM1846 | *MAT a CHS2-3MYC::TRP* | This Study |
| FM2663 | *MAT a CHS2-3MYC::TRP end3::natNT2* | This Study |
| FM3150 | *MAT a CHS2-3MYC::TRP sla2::natNT2* | This Study |
| FM3369 | *MAT a GFP-TUB1::URA MYO1-GFP::TRP NDC10-tdtomato::loxp-KAN-loxp* | This Study |
| FM3382 | *MAT a GFP-TUB1::URA MYO1-GFP::TRP NDC10-tdtomato::loxp-KAN-loxp end3::natNT2* | This Study |
| FM2642 | *MAT a MYO1-GFP::KAN GFP-TUB1::URA* | This Study |
| FM3046 | *MAT a MYO1-GFP::KAN GFP-TUB1::URA ede1::natNT2* | This Study |
| FM3260 | *MAT a MYO1-GFP::KAN GFP-TUB1::URA sla2::natNT2* | This Study |
| FM2665 | *MAT a MYO1-GFP::KAN GFP-TUB1::URA end3::natNT2* | This Study |
| FM3262 | *MAT a MYO1-GFP::KAN GFP-TUB1::URA rvs161::HIS rvs167::natNT2* | This Study |
| FM4525 | *MAT a MYO1-GFP::TRP GFP-TUB1::URA ADH1-yeOsTIR1::LEU* | This Study |
| FM4705 | *MAT a MYO1-GFP::TRP GFP-TUB1::URA ADH1-yeOsTIR1::LEU end3::natNT2* | This Study |
| FM4543 | *MAT a MYO1-GFP::TRP GFP-TUB1::URA ADH1-yeOsTIR1::LEU CHS2-1x Mini-AID::KAN* | This Study |
| FM4549 | *MAT a MYO1-GFP::TRP GFP-TUB1::URA ADH1-yeOsTIR1::LEU CHS2-1x Mini-AID::KAN end3::natNT2* | This Study |
| FM2784 | *MAT a MYO1-GFP::KAN GFP-TUB1::URA SPC29-RFP::hphNT1* | This Study |
| FM2908 | *MAT a MYO1-GFP::KAN GFP-TUB1::URA SPC29-RFP::hphNT1 ede1::natNT2* | This Study |
| FM2910 | *MAT a MYO1-GFP::KAN TUB1-GFP::URA SPC29-RFP::hphNT1 sla2::natNT2* | This Study |
| FM2817 | *MAT a MYO1-GFP::KAN GFP-TUB1::URA SPC29-RFP::hphNT1 end3::natNT2* | This Study |
| FM3224 | *MAT a MYO1-GFP::KAN GFP-TUB1::URA SPC29-RFP::hphNT1 rvs167::natNT2 rvs161::HIS* | This Study |
| FM3285 | *MAT a MYO1-GFP::KAN GFP-TUB1::URA SPC29-RFP::hphNT1 fks1::LEU* | This Study |
| FM3296 | *MAT a MYO1-GFP::KAN GFP-TUB1::URA SPC29-RFP::hphNT1 fks1::LEU end3::natNT2* | This Study |
| FM3227 | *MAT α GFP-TUB1::URA MYO1-GFP::KAN chs3::KAN SPC29-RFP::hphNT1* | This Study |
| FM3264 | *MAT α GFP-TUB1::URA MYO1-GFP::KAN chs3::KAN SPC29-RFP::hphNT1 end3::natNT2* | This Study |
| FM3349 | *MAT a MYO1-GFP::KAN GFP-TUB1::URA SPC29-RFP::hphNT1 GAL-CHS2-13MYC::TRP chs2::HIS* | This Study |
| FM3200 | *MAT a MYO1-GFP::KAN GFP-TUB1::URA SPC29-RFP::hphNT1 GAL-CHS2-13MYC::TRP chs2::HIS end3::natNT2* | This Study |

*natNT2* refers to Nourseothricin

*hphNT1* refers to Hygromycin B
